# Feeding sows milk biofortified with n-6 and n-3 modulates immune status of sows and drives positive transgenerational effects

**DOI:** 10.1371/journal.pone.0306707

**Published:** 2024-08-27

**Authors:** Leriana Garcia Reis, Vera Letticie de Azevedo Ruiz, Simone Maria Massami Kitamura, André Furugen Cesar Andrade, Fernando de Oliveira Bussiman, Mirele Daiana Poleti, Juliano Coelho da Silveira, Heidge Fukumasu, Lúcia Helena Faccioli, Cleni Mara Marzocchi-Machado, Ricardo de Francisco Strefezzi, Edna Neves Garcia, Theresa Casey, Arlindo Saran Netto

**Affiliations:** 1 Department of Animal Science, Purdue University, West Lafayette, Indiana, United States of America; 2 Department of Veterinary Medicine, School of Animal Science and Food Engineering, University of São Paulo, Pirassununga, SP, Brazil; 3 Department of Animal Science, School of Animal Science and Food Engineering, University of São Paulo, São Paulo, Brazil; 4 Department of Animal Reproduction, School of Animal Science and Food Engineering, University of São Paulo, São Paulo, Brazil; 5 Department of Animal and Dairy Science, University of Georgia, Athens, Georgia, United States of Ameirca; 6 Department of Clinical Analyses, Toxicology and Food Sciences, School of Pharmaceutical Sciences of Ribeirão Preto, University of São Paulo, Ribeirão Preto, SP, Brazil; University of Life Sciences in Lublin, POLAND

## Abstract

The risk of chronic diseases such as cardiovascular disease, cancer, inflammation, obesity, and autoimmune disease is linked to the quality of dietary fats, with lower intake of saturated and higher intake of n-6 and n-3 polyunsaturated fatty acids (PUFA) considered beneficial to health. This study investigated the effect of supplementing sows’ diets with cow’s milk biofortified with n-6 or n-3 PUFA, at varying n-6/n-3 ratios (8.26, 7.92, and 2.72) during their growing phase and throughout gestation and lactation on their reproductive performance and immune-inflammatory status. Specifically, we analyzed circulating cholesterol and fatty acid profiles of serum, colostrum and milk, sow body weight, and neonate colostrum intake, Apgar scores, muscle composition, and embryo viability. Analysis of circulating immunoglobulins (Ig), interleukins, and eicosanoids and complement system hemolytic activity were used to evaluate inflammatory and immune responses of sows and piglets. Expression of lipolysis and lipogenic genes in the liver were investigated in sows and piglets, with additional investigation of hypothalamus genes regulating appetite in sows. Feeding sows milk biofortified with n-6 and n-3 PUFA altered serum fatty acid profiles, reduced triglycerides (TG), increased embryo total number, increased early gestation backfat, and reduced colostrum IgG. Piglets of biofortified sow had higher circulating IgA, IgM and TNF-α, and lower IL-10. Sows fed n-3 biofortified milk had higher very low-density lipoproteins (VLDL) and TNF-α in circulation. Offspring from sows fed n-6 versus n-3 biofortified milk had lower IL-10 and expression levels of SREBP-1. N-3 versus n-6 also lowered arachidonic acid (ARA) levels in sow’s milk and piglet viability 1. Findings offer insights into the potential health benefits of dietary supplementation with biofortified milk in swine, which serve as good model of diet-nutrition studies of humans, and therefore can potentially be considered in dietary recommendations both human and animal populations.

## Introduction

Dietary fats play a diverse and vital role as a concentrated source of energy and in supporting numerous essential physiological and molecular functions, including the absorption of fat-soluble vitamins, regulation of inflammatory responses and cellular signaling, and the formation of biological membranes [1]. Some common sources of polyunsaturated fatty acids (PUFAs) in animal feed are vegetable oils like soybean oil, corn oil, and sunflower oil, which are rich sources of linoleic acid (LA), and fish oil, linseed, algal oil, marine algae, canola oil are rich in alpha-linolenic acid (ALA) [2–4]. LA and ALA serve as precursors to the n-6 and n-3 families of PUFA such as γ-Linolenic (18:3 n-6), dihomo-γ-linolenic (20:3 n-6), arachidonic acid (ARA) (20:4 n-6), dihomo-α-linolenic (20:3 n-3), eicosapentaenoic (EPA) (20:5 n-3) and docosahexaenoic acid (DHA) (22:6 n-3), which mediate metabolic functions, inflammatory responses and serve as integral membrane components of the central nervous system [5, 6]. Over the last several decades a disequilibrium in the proportion of LA to ALA evolved in modern Western diets changing from the recommended 1:1, 5:1 and 10:1 ratio, to 20:1 or over 50:1 in n-6/n-3 ratio.

Dietary intake and nutritional state beginning prior conception and throughout gestation and lactation influence coordinated maternal physiological and behavioral adaptations to these states, which create the metabolic and immunologic environment for development, and growth of offspring [7, 8]. Studies of the impact of varying n-6/n-3 ratio in maternal diet indicate it impacts both the mother and her offspring. Incorporating different source of lipids into the sow diet is recognized to alter the fatty acid in blood circulation affecting organ functions, consequently influencing the composition of milk, such as increased omega-6 acid intake diminishes muscle insulin sensitivity while fostering fat accumulation in adipose tissue. Whereas dietary strategies incorporating omega-3 fatty acids counteract this dysregulation, enhancing insulin sensitivity and regulating body fat [9]. Therefore, fatty acids dietary modification has the potential to enhance piglet growth by increasing energy availability [10]. This effect extends from the prenatal phase, aiding in fetal formation, to postnatal stages, facilitating growth through increased energy provision and interactions in protein synthesis [11].

Studies have found that feeding lactating sow sunflower oil led to a high n-6/n-3 PUFA ratio and altered the fatty acid profile of immune cells in their offspring showed high levels of macrophage synthesis and of the inflammatory mediators prostaglandin E2 and thromboxane B2 [12]. Researchers observed that varied ratios of n-6/n-3 PUFA in sows by supplementing diets with soybean oil (13:1 or 10:1) or linseed oil (4:1) throughout the gestation and lactation periods revealed that the low n-6/n-3 PUFA maternal diet improved weaning survival rate, weight gain in suckling piglets, and increased levels of total n-3 fatty acids in colostrum and milk [13]. In another study, In another study, the low ratio n-6/n-3 diet of 4:1 throughout the gestation and lactation periods was correlated with higher piglet birth weight and fat deposition in suckling piglets [14, 15]. Proteome analysis of piglet muscle and fat tissue indicated a negative change in the immune status of piglets when sows were fed higher n-6/n-3 PUFA diets, reflected by higher abundance of haptoglobin, an acute-phase protein, and the activation of greater abundance of proteins associated with the innate immune response and acute inflammatory response [14], which may be linked to the pro-inflammatory characteristics of n-6 PUFAs. Although, these findings indicate that maternal diets varying in n-6/n-3 ratio affect offspring growth, development and health, the matrices of the fats varied between the diets, and thus findings may not be related to the ratio, rather other aspects of the oils added to maternal diets. Moreover, the length of supplementation in diets varied among the studies.

Furthermore, lipids have garnered attention for their involvement in gene expression linked to various health conditions such as inflammation and obesity. The dietary choices we make exert a profound influence on the body’s physiological functions. Additionally, the prenatal environment significantly shapes fetal development, influencing susceptibility to obesity and related metabolic disorders, such as glucose intolerance and hypertension, in later life [16]. Polyunsaturated fatty acids, particularly those from the n-3 series, have emerged as key players in disease prevention. They modulate membrane composition, cellular metabolism, signaling pathways, and gene expression. PUFAs exert their regulatory effects on gene expression across different tissues, including the liver, heart, adipose tissue, and brain, mediated by transcription factors like SREBP-1c (Sterol Regulatory Element-Binding Protein 1c) and nuclear receptors such as PPAR-α (Peroxisome Proliferator-Activated Receptor α) [17]. Moreover, PUFAs exert control over fatty acid biosynthesis rates by inhibiting hepatic lipogenesis through the suppression of acetyl-CoA carboxylase (ACC) and fatty acid synthase (FAS) [18, 19].

Over the past decade, extensive research has delved into the genetic mechanisms governing appetite regulation within the hypothalamus. Studies have illuminated that supplementing expectant mothers with n-3 polyunsaturated fatty acids (PUFAs) during pregnancy, achieving a lower ratio of n-6 to n-3 PUFAs, typically decreases leptin expression (LEPR) and circulating levels. This observed effect is attributed to epigenetic alterations of the gene promoter, both in animal models and human offspring, suggesting promising long-term health benefits. However, it is significant that discrepancies exist in the findings across studies [20–24]. Moreover, recent investigations underscore the diverse beneficial biological effects of n-3 PUFAs, including their potential in enhancing insulin resistance. Particularly significant is the pivotal role played by an important shift in the n-6/n-3 ratio towards n-3 PUFAs in the prevention and management of metabolic disorders, such as metabolic syndrome [25, 26].

We recently developed an approach to biofortify milk with n-6 and n-3 PUFA by feeding Holsteins cows with linseed and soybean oil, respectively [27]. Holstein cows supplemented with soybean oil and linseed oil resulted in milk with ratios of n-6/n-3 PUFA of 8.26 and 2.72, respectively, referred to as n-6 milk and n-3 milk. Milk linseed oil supplemented cows reduce thrombogenicity index and atherogenicity index and the ratio of hypocholesterolemic to hypercholesterolemic fatty acids increased [9]. Therefore, alterations of fatty acid profiles of biofortified n-3 and n-6 milk reflect a healthier milk fat profile for human consumption.

Pigs serve as a valuable animal model for human research due to their anatomical and physiological similarities to humans, such as size, immunology, genome, and physiology [28]. Greater similarities between pigs and humans than humans with rats and mice, make pigs preferable to rodents for translational and clinical research [29]. Pigs are extensively used to study embryonic development and immune responses due to comparable development and responses of humans [30]. Previous studies from our group showed that the biofortified n-3 and n-6 milk, lead to alterations of fatty acid profiles reflect a healthier milk fat profile for human consumption and lower n-6/n-3 ratio was observed in n-3 supplemented sows. Also, the biofortified n-3 milk reduced stearic acid (18:0), supporting a favorable PUFA profile in diet positively impacts metabolic phenotype of recipient [31]. Piglets from biofortified milk-fed sows were lighter at birth but gained more weight from 1 to 21 days old, with higher levels of EPA in their serum, leading to a reduced ARA/EPA ratio compared to the control group [32].

The authors pursued the particular research goal because of the critical role dietary fats play in human health and disease risks. Epidemiologic studies have linked the quality and types of fats in diets with health and disease risks. Diets high in saturated fatty acids (SFA) are associated with increased risk of obesity, diabetes, cardiovascular disease, cancer, and inflammatory and autoimmune diseases [33]. Therefore, the general dietary recommendations are to reduce saturated fatty acid intake and to ensure adequate intake of the essential polyunsaturated fatty acids (PUFA), as linolenic acid (LA, 18:2 n-6) and α-linolenic acid (ALA, 18:3 n-3). Over the last several decades a disequilibrium in the proportion of LA to ALA evolved in modern Western diets changing from the recommended 1:1, 5:1 and 10:1 ratio, to 20:1 or over 50:1 in n-6/n-3 ratio. High ratio of n-6/n-3 intake is associated with inflammation and the same chronic diseases linked to a high level of saturated fatty acid intake. There is thus a need for relevant information regarding the ideal balance of n-6 and n-3 lipids in diets for health, growth, and development, for both, human and pig production, since the similarities of their organism.

The overall aim of the current study was to determine if PUFA biofortified milk affected reproductive competence of sow as assessed by number of embryos, vigor of neonates at birth, colostrum production and intake, as well as sow backfat depth and biochemical parameters across gestation and lactation to better understand factors that may underlie observations related to better growth rates in neonates during lactation as well as investigated whether biofortified milk altered inflammatory and immune responses in sows and their offspring. Moreover, we investigated whether changes in circulating fatty acid profiles related to differences in expression of genes that regulate fatty acid metabolism in the liver of sows and piglets and whether expression of genes that control appetite in the arcuate nucleus of the hypothalamus differed in sows during the gestation period.

## Material and methods

All activities performed in this study were reviewed and approved by the Animal Care Committee of the Faculty of Animal Science and Food Engineering, University of São Paulo (Protocol #4939070317 and #6983020920) before commencing.

### Animals, facilities, and treatment allocation

**Fig 1:**
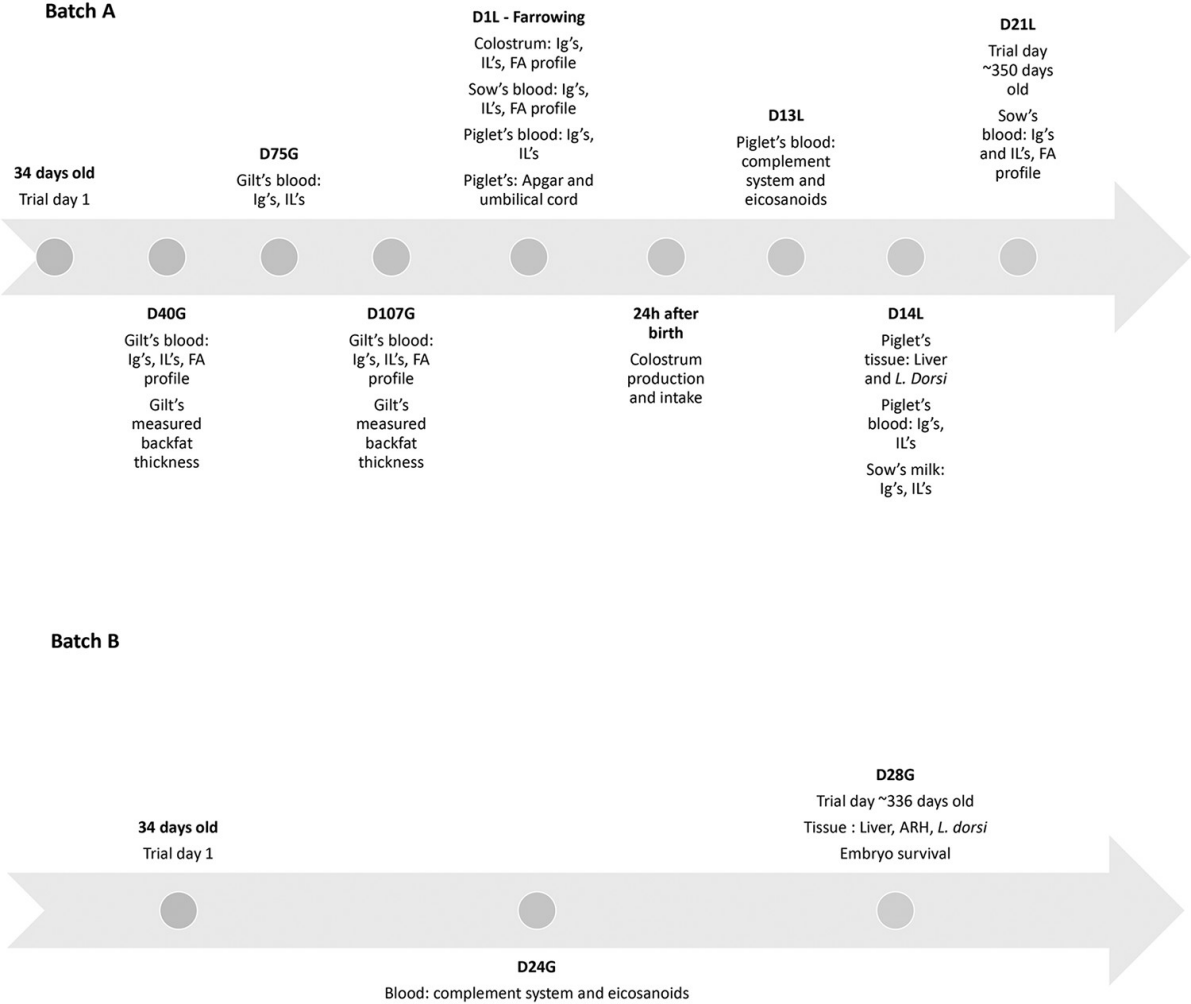
Timeline of point collections for swine females and piglets. D24G, day 24 of gestation; D28G, day 28 of gestation; D40G, day 40 for gestation; D75G, day 75 for gestation; D107G, day 107 for gestation; D1L, day 1 of lactation/day of farrowing; 24h after birth, day after the farrowing; D13L, day 13 of lactation; D14L, day 14 of lactation; D21L, day 21 of lactation.

Sixty sows, including thirty F1 generation sows (Landrace x Large White, at 34 d of age, weighing 9.59 ± 1.28 kg) referred as batch A, and thirty F2 generation sows (Landrace x Large White, 30 ± 2 days weighing 8.49 ± 1.81 kg) referred as batch B, were followed through growth phases, puberty, breeding, gestation, and lactation d 21. Batch A was evaluated until lactation d 21 and batch B was euthanized on gestation day 28 ± 3 for embryo vitality evaluation (Fig 1). For each batch, sows were randomly assigned to one of three treatment groups: control (**CON**, n = 20), basal diet + milk from cows without oil supplementation, omega-3 (**n-3**, n = 20), basal diet + milk from cows fed an enriched diet with linseed oil, and omega-6 (**n-6**, n = 20), basal diet + milk from cows fed an enriched diet with soybean oil (see (27) for approach to feeding cows and composition of biofortified and control milk). No criteria were established a priori for including and excluding animals (or experimental units) during the experiment.

Sows were individually housed in crates providing 0.75 m^2^/animal from 34 to 76 days of age. Subsequently, they were to crates providing 2.06 m^2^/animal, including gestation and lactation cages, from 77 days of age until the end of experiment. The experimental animals were fed for *ad libitum* intake twice daily (at 07:00 am and 1:00 pm) during the grower phase (30–130 days of age). After this period, the composition and nutritional level of diets met the Rostagno-recommended requirements [34]. Water was always available *ad libitum*. Sows from batch A were transferred onto the lactation diet and fed for *ad libitum* intake. Sows from batch B were fed with 2 kg/d of gestation diet from d 21 of gestation until the day of slaughter. The diets were based on corn and soybean feedstuffs and did not contain any oil as an ingredient (Table 1).

**Table 1:** Composition of pre-initial, initial, growth, finisher, replacement, gestation, and lactation diets, specific to each physiological phase of swine female.

Item	Pre-initial	Initial	Grower	Finisher	Replacement	Gestation	Lactation
*Ingredients*, *g*.*kg*^*-1*^							
Ground corn	399.0	649.0	699.0	739.0	644.4	594.0	587.8
Soybean meal	200.0	300.0	280.0	240.0	240.8	140.0	265.1
Wheat bran	-	-	-	-	86.5	240.0	-
UNIMIX^*a*^	400.0	50.0	20.0	20.0	25.0	25.0	30.0
L-lysine	-	-	-	-	-	-	10.0
DL-methionine	-	-	-	-	-	-	2.0
Sugar	-	-	-	-	-	-	100.0
Calcitic limestone	-	-	-	-	2.3	-	4.1
Mycofix	1.0	1.0	1.0	1.0	1.0	1.0	1.0
*Chemical composition*							
Dry matter, %	83.41	87.92	87.07	88.93	88.12	89.42	88.25
Ashes, %	6.69	5.43	4.80	3.70	4.54	5.23	5.62
Crude energy, cal.g^-1^	4347.0	4393.0	4419.0	4465.0	4411.5	4360.0	4376.0
Ether extract, %	4.14	1.71	2.41	1.71	1.37	1.40	1.56
Crude fiber, %	4.54	4.32	4.30	4.07	3.01	5.23	5.28
Crude protein, %	18.04	21.21	18.07	20.46	19.04	17.59	19.99
Calcium, %	0.90	0.80	0.63	0.51	0.82	0.81	0.94
Phosphorus, %	0.69	0.53	0.41	0.37	0.38	0.46	0.43

^a^The premix used in each phase was based on Rostagno et al. [34]. The guaranteed levels per kilogram of the product are found in S2 File. Pre-initial: from 34 days to 42 days, Initial: from 43 days to 76 days, Grower: from 77 to 128 days, Finisher: from 129 to 174 days, Replacement: 175 to 190 days, Gestation: from the 21^st^ of pregnancy until the day of birth, Lactation: from the 1^st^ day of lactation until weaning.

Supplementation with control and biofortified milks, n-3 and n-6, was provided at approximately 5 mL/kg body weight met the IBGE-recommended requirements [35]. Milk supplements were administered to pigs at 08:00 am, after individual feeding. Each sow was supplemented individually in the feeder with 200 mL (from 34 or 30 ± 2 days to 76 days), 300 mL (from 77 to 128 days), 400 mL (from 129 to 174 days), 500 mL (175 to 190 days) and 1 L (over 190 days) of cow’s milk by the end of the experiment. The biofortified milk provided to the sows in this current study was obtained from the study carried out by Oliveira et al. [27] using Holstein cows. Briefly, the cows were supplemented or not with 2.5% (dry matter basis) of linseed or soybean oil, sources of n-3 and n-6, respectively. The effects of these vegetable oil inclusion on the lipid fraction profile of dairy cows’ milk are presented in Table 2.

**Table 2:** Composition of lipids in biofortified cow’s milk by saturation level, n-6 and n-3 content, and cholesterol concentration.

Fatty acid profile^*a*^, g.100 g^-1^	Diets^*b*^			SEM^c^		*P* ^*d*^	
	CONTROL	LIN	SOY		Treatment	C1	C2
ΣSFA	66.887	56.605	56.524	1.441	<0.01	<0.01	0.969
ΣUSFA	33.051	43.346	43.394	1.438	<0.01	<0.01	0.986
SFA/USFA	2.121	1.364	1.341	0.107	<0.01	<0.01	<0.01
ΣMUFA	29.583	39.470	39.551	1.301	<0.01	<0.01	0.966
ΣPUFA	3.570	3.979	3.935	0.275	0.199	0.076	0.860
Σn-3	0.324	1.022	0.360	0.029	<0.01	<0.01	<0.01
Σn-6	2.483	2.252	2.880	0.208	0.004	0.589	0.001
n-6/n-3	7.919	2.724	8.263	0.504	<0.01	<0.01	<0.01
Cholesterol, g.100 mL^-1^	9.942	8.741	10.053	1.649	0.174	0.403	0.097

^a^ΣSFA = Σ saturated fatty acids; ΣUSFA = Σ unsaturated fatty acids; SFA/USFA = Σ saturated/Σ unsaturated; ΣMUFA = Σ monounsaturated fatty acids; ΣPUFA = Σ polyunsaturated fatty acids; Σ n-3 = Σ omega-3 fatty acids; Σ n-6 = Σ omega-6 fatty acids; n-6/ n-3 = Σ omega-6/Σ omega-3.

^b^Cows fed with a control diet (CONTROL), supplemented with linseed oil (LIN) or soybean oil (SOY).

^c^SEM, standard error of the means.

^d^C1, contrast between CONTROL vs LIN+SOY; C2, contrast between LIN vs SOY.

### Reproductive management

Estrus among sows was synchronized using 5 mL of altrenogest (Regumate®, Intervet Inc., MSD Animal Health, São Paulo, Brazil) per animal for 18 consecutive days and estrus was checked twice daily in the presence of a mature boar. Sows were artificially inseminated at 198 ± 9 days of age using refrigerated semen from hybrid boars (DB LM6200, Patos de Minas, Brazil). They were inseminated at first signs of standing estrus and every 24 h while exhibiting signs a standing estrus. Seminal doses were stored at 17°C and used within 3 days, with a final concentration of 30 × 10^6^ sperm cell/mL (>80% motility, 100 mL per seminal dose). Pregnancy was confirmed through ultrasound examination (Pie-Medical Scanner 100, 3.5 MHz convex probe, Philipsweg, Belgium) on 21 d post-insemination.

### Collection time points

Blood samples from sows in batch A were collected at D40G (day 40 of gestation), D75G (day 75 of gestation), D107G (day 107 of gestation), D1L (day 1 of lactation day, also day of farrowing), D13L (day 13 ± 4 of lactation) and D21L (day 21 of lactation). To determine body condition during gestation, backfat thickness (BF) was evaluated on days D40G and D107G. Apgar and umbilical cord scores were evaluated at D1L in their piglets; the evaluation of colostrum production and intake occurred 24 h after birth. Colostrum was collected in batch A on D1L and milk was collected at D14L. The piglets’ blood was collected at D1L, D13L and D14L. Four piglets per sow (2 males and 2 females) were euthanized by electrocution followed by exsanguination and liver and *Longissimus dorsi* muscle were collected at D14L (day 14 of lactation).

Blood samples from sows in batch B were collected on D24G (day 24 ± 6 of gestation). Those females were euthanized at D28G (day 28 ± 3 of gestation) by electrocution followed by exsanguination, embryos were counted, *Longissimus dorsi* muscle, liver, and arcuate nucleus of the hypothalamus (ARH) were collected and snap frozen (Fig 1). The 28th day of pregnancy was chosen as the moment of collecting fetuses to count the number of viability because it falls within a critical period of gestation where fetal development and viability can be assessed, once the conceptuses that die between days 12 and 45 of pregnancy are completely reabsorbed by the uterus. Thus, this timing allows for the differentiation between early embryonic losses and losses occurring later in gestation [36].

### Blood collection

Blood samples were collected by jugular venipuncture using 18 g x 1.5-inch needle for sows, 21 g x 1.5-inch needle (Becton Dickinson & Company, Juiz de Fora, MG, Brazil) for piglets. A 3 mL serum Vacuette^®^ (Greiner Bio-One Brasil Produtos Médicos Hospitalares Ltda, Americana, SP, Brazil) tube was used to collect blood to measure fatty acids, biochemical parameters, and activity of complement system. Blood was collected into a 3 mL EDTA Vacuette^®^ (Greiner Bio-One Brasil Produtos Médicos Hospitalares Ltda, Americana, SP, Brazil) tube for plasma analysis of immunoglobulin and interleukin, and a 3 mL heparin Vacuette^®^ (Greiner Bio-One Brasil Produtos Médicos Hospitalares Ltda, Americana, SP, Brazil) tube was used to collect blood for plasma analysis of eicosanoids. Blood was a lowed to clot at room temperature for serum isolation, which was collected following centrifugation for 10 min at 2,000 g ~25°C. Plasma and serum were stored at -80°C until analysis.

### Performance parameters

Individual sows in batch A were weighed and scanned for BF on D40G and D107G to determine changes in body weight (BW) and BF across gestation. The BF of the sows was measured at the P2 position (5 cm off the midline at the 10th rib) with ultrasound instrument (Pie-Medical Scanner 100, 3.5 MHz convex probe, Philipsweg, Belgium).

### Determination of fatty acids profile and biochemical parameters

The sow’s serum FA profile was analyzed by gas chromatography [37], on samples collected on D40G, D107G, D1L and D21L. Serum levels of urea, total protein, total cholesterol, high-density lipoprotein (HDL), low-density lipoprotein (LDL), very low-density lipoprotein (VLDL), glucose, and triglycerides (TG) were determined in the Clinical Analysis Diagnostics laboratory (DAC, Pirassununga, SP, Brazil) using commercially available kits (VIDA Biotecnologia® and LABTEST®, Minas Gerais, Brazil) according to the manufacturer’s instructions.

### Colostrum and milk samples

Colostrum samples were manually collected (20 mL) during farrowing when oxytocin is naturally high. Milk samples (50 mL) were obtained on D14L following administration of 1 mL of oxytocin (Ocitovet, Ceva, Brazil) through the sows’ ear vein. All samples were obtained by manually milking from all teats and combined to create a uniform sample. Samples were stored at −20°C until they were analyzed. For quantification of immunoglobulins and interleukins, all colostrum and milk samples were thawed in ambient temperature and centrifuged at 10,000 x g for 5 min to remove fat.

### Measure of colostrum production and intake

Colostrum intake (CI) of piglets was estimated using the model developed by Theil et al. [38] 24 h after the first-born piglet. CI and weight gain (WG) were expressed in grams, body weight at birth (BWB) in kilograms and duration of CI in minutes (D):

CI = –106 + 2.26WG + 200BWB + 0.111D – 1,414WG/D + 0.0182WG/BWB

According to Devillers et al. [39] a negative CI was assumed to be 0. The sum of the individual CI of each piglet within the litter was used to calculate colostrum production of the sow [38].

### Apgar score and umbilical cord evaluation

Neonates were evaluated at birth for vitality by three investigators previously trained using the Apgar score described by our colleagues [40], adapted by the authors [41] and modified later [42]. In order to avoid interference with vitality criteria, no aid was given to newborn piglets at the time of farrowing and during the vitality evaluation.

The following variables were quantified: time from birth to breathing: >1 min, between 16 s and 1 min and <15 s; heart rate: bradycardia (<120 bpm), normal (between 121 and 160 bpm) and tachycardia (>161 bpm); meconium stain: severe, mild or absent [43]; color of the skin on the snout: cyanotic, pale or pink; and attempts to stand on all four legs: >5 min, between 1 and 5 min and <1 min. The score for each variable was from 0 (the least favorable) to 2 (the most favorable) and the sum of them in an Apgar score ranging from 1 to 10 for each neonatal piglet. All piglets were classified into three groups of Apgar scores, within each litter: low vitality (scores ≤5); medium vitality (scores between 6 and 7); and high vitality (scores ≥8) [44]. Since scores ≥6 means medium to high vitality, lower scores are of concern regarding neonate survival. Thus, piglets with Apgar vitality scores between 1 and 5 were classified in the statistical analysis as viability 1, i.e., representing low survival, in view of the interest in verifying the potential of biofortified milk to reduce low survival at birth. Umbilical cords were evaluated at birth and classified as adhered (normal) or broken (abnormal) according to the criteria from [45].

Heart rates (beats/min) were measured by use of a stethoscope, and first inspiratory efforts were considered when a movement was observed in the thoracic area accompanied by exhalation of air. The time until each newborn was able to stand on all four feet was measured with a chronometer. In addition, the first mammary contact was recorded through individual piglet observation, independently if there was colostrum consumption or not, from the moment the newborn was relocated close to the mother’s vulva. At the end, all procedures were carried out within 5 min, to shorten the handling duration. All the piglets were individually identified with numbers on their backs and subsequently with ear tags.

### Analysis of embryo viability

At day 28 ± 3 of gestation, sows were humanely slaughtered, (at University of São Paulo) through electrocution and exsanguination. The entire reproductive tract was removed, and the pregnant uterus weigh was recorded. The tract was open, and the number of embryos counted and categorized as viable or nonviable. The number of corpora lutea was counted and the fertilization rate was determined by dividing the number of total embryos by the number of corpora lutea. The adjusted fertilization rate was calculated using number of viable embryos compared with the total number of corpora lutea counted.

### Histological analysis

*Longissimus dorsi* muscle samples were collected on D28G (F2 sows) and (D14L) (F1 offspring) and fixed in 10% formalin for 24 h and stored in 70% alcohol until embedding in paraffin. Sections (4 μm) were mounted on glass slides and stained with hematoxylin and eosin, as previously described [46].

Three random areas (40x) of each slide were captured as image for each location (muscle and intramuscular adipocytes), using a microscope Leica DM500 connected a camera Leica ICC50-HD (Leica Microsystems, Heerbrugg, Suíça). The images were evaluated by an image analysis software (Image-Pro Plus®, Media Cybernetics, Rockville, USA), to determine through Manual Tag tool: area, maximum diameter, minimum diameter, and average diameter of muscle and adipocytes. The per-area is a percentage of occupied area by delimited object inside to the total image. This is a way to quantify the area of relative form. The morphometric analysis was performed blindly, and cells were chosen with good boundaries cytoplasmic. The aim was to verify if differences in physical structure of lipids on diet in the long-term could modulate the development of muscle and adipocytes.

### Evaluation of gene expression by qRT-PCR analysis

On D28G (F2 sows) and D14L (F1 offspring), liver samples were immediately placed in cryotubes with RNAlater™ Stabilization Solution (InvitrogenTM, AM7024, Carlsbad, CA, USA) and snap frozen in liquid nitrogen. Meanwhile, ARH samples were swiftly subjected to direct snap freezing in liquid nitrogen. Samples were stored at -80°C until analysis.

The primers were designed using FASTA sequence files accessed through the National Center for Biotechnology Information (NCBI) GenBank database of *Sus scrofa* published nucleotides sequences using NetPrimer Software (PREMIER Biosoft International, Palo Alto, CA, USA; Tables 1 and 2 in S1 File).

Total RNA was extracted using RNeasy Mini Kit (Qiagen, #74104, Venlo, Limburg, Netherlands) according to the manufacturer’s instructions. The quantity of the RNA was determined using NanoDropOneC spectrophotometer (Thermo Scientific, Carlsbad, CA, USA), and the integrity was assessed by visualization of the 28S/18S rRNA band pattern in a 1% agarose gel. Isolated RNA was treated with DNAse I (Thermo Scientific, Carlsbad, CA, USA) to eliminate possible genomic DNA contamination. The synthesis of cDNA was performed using the High-Capacity cDNA Reverse Transcription Kit (Applied BiosystemsTM, #4368814, Foster City, CA, USA), according to the manufacturer’s protocol. Real-Time Quantitative Reverse Transcription PCR (qRT-PCR) analyses of mRNAs were carried out in 10 μL reactions containing 2x qPCRBIO SyGreen Mix (PCRBiosystems, London, UK), 1 μl cDNA (200 ng/μL), and forward and reverse primers in optimized concentrations determined for each gene with the following cycling parameters: 2 min at 95°C followed by 40 repeats of 5 s at 95°C and 30 s at 60°C followed by melt curve analysis to confirm amplification of single cDNA products. Relative quantitative gene expression was calculated to the transcript level of hypoxanthine phosphoribosyltransferase 1 (HPRT1) and β-actin (ACTB) (housekeeping genes), as previously described [47, 48]. Raw Ct values were normalized to the geometric average of the reference genes HPRT1 and ACTB as previously described for similar tissues [49, 50]. Normalized results of duplicate samples were used to calculate the relative expression using 2^-ΔCt^ transformation and the relative expression values were calculated using the ΔCt method [51].

### Hemolytic activity of the complement system

Sow (batches A and B) and piglet serum samples were used to measure the hemolytic activity of the complement system as described [52] by a kinetic test in Department of Clinical Analyses, Toxicology and Food Sciences, School of Pharmaceutical Sciences of Ribeirão Preto, University of São Paulo, Brazil. Erythrocytes from rabbit peripheral blood, collected in Alsever’s solution as an anticoagulant, were added with 10mM TEA-EDTA solution containing 0.1% gelatin. The mixture was kept at 37°C for 15 minutes. After centrifugation at 480 x g at 4°C for 10 minutes, the supernatant was discarded, and the erythrocytes were washed three times in 2mM TEA-MgSO_4_ solution before use. An aliquot of this suspension was prepared in a 2mM TEA-EGTA-MgSO_4_ solution containing 0.1% gelatin and adjusted to an optical density between 0.8 and 0.9 at 700nm when in the final volume of the assay with pig serum. The diluted serum was added to the erythrocyte suspension and read at 700 nm for 15 min at 37°C to evaluate complement hemolytic activity (Epoch 2; BioTek Instruments, Inc., Winooski, VT, USA). Preheated serum at 56°C for 30 minutes was used as a negative control for complement activity. The calculation of 50% hemolysis was derived from hemolysis curves obtained by diluting serum from female pigs. This involved determining the T1/2 value, which represents the time required for complement to reduce the initial optical density of the rabbit erythrocyte suspension by 50%. For piglet serum, hemolytic activity was calculated by the ΔOD value, which is the difference between the final and initial optical densities.

**Fig 2:**
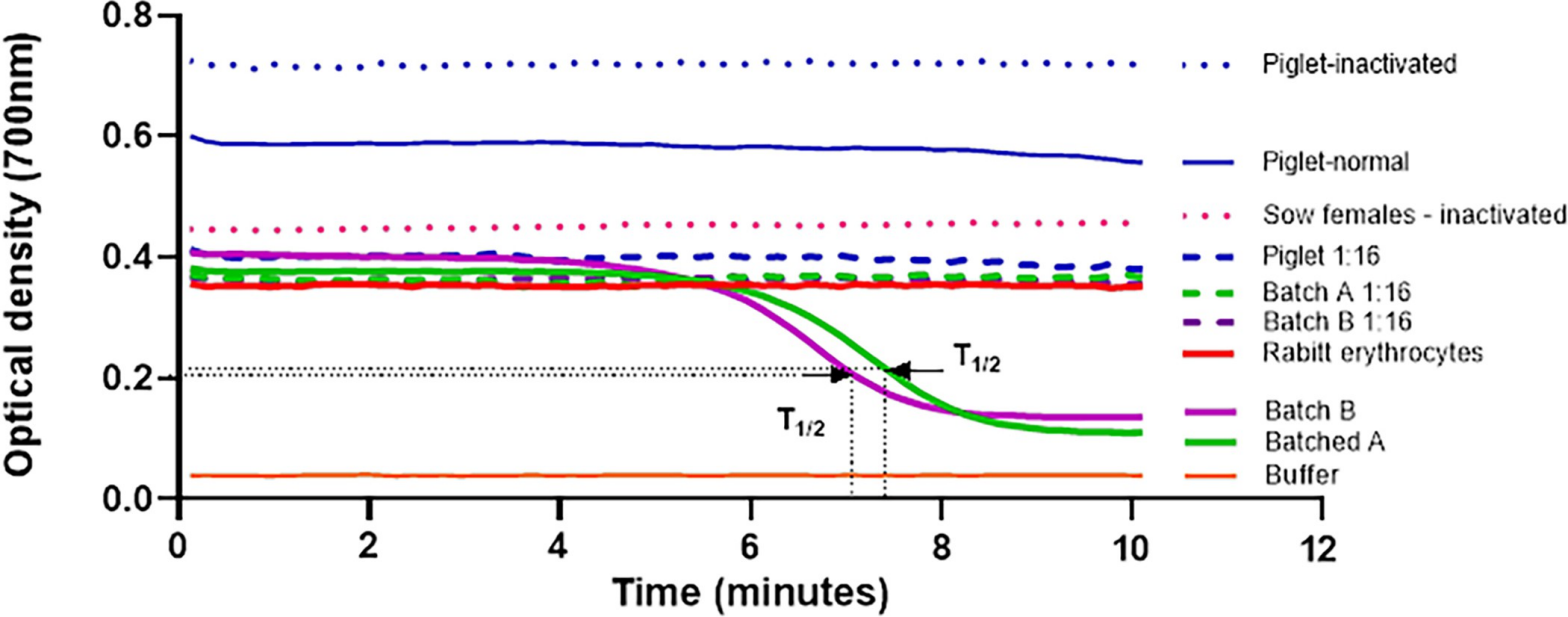
Representation of the lysis curves obtained to evaluate the hemolytic activity of the alternative pathway of the complement system. Pooled samples of serum from the experimental groups were tested undiluted and diluted 1:16. The experimental controls were pooled inactivated serum from the respective experimental groups, buffer in the absence of serum and erythrocytes, and rabbit erythrocytes in the absence of serum. Arrows indicate T1/2 values for serum pools from batches A and B.; T1/2, time required for complement to reduce 50% of the initial optical density of the rabbit erythrocyte suspension.

The Fig 2 represents the lysis curve achieved for standardization of trial. The serum samples of experimental groups: control, batch A, batch B and piglets were used in this standardization. Each experimental group, pooled of serum was prepared and submitted to test undiluted or diluted 1:16. Thus, from the lysis curves the value of T1/2 was calculated. These values account for the hemolytic activity per sample, in other words, the lower T1/2, the higher hemolytic activity of complement.

The outcomes presented in Fig 2 are T1/2 values to hemolytic activity of complement in serum samples of batches A and B were determined undiluted, also indicated by the arrow. However, were not observed hemolytic activity to dilutions 1:16. As to the pooled of serum piglet samples the T1/2 values were not determined to sample undiluted or 1:16. In this case, the calculations will be based on variation between initial and final optic density (ΔOD).

### Quantification of immunoglobulins and interleukins

Enzyme-linked immunosorbent assay (ELISA) kits were used to measure levels of IgA, IgM, IgG (Bethyl Laboratories, Montgomery, TX, USA), IL-10, TNF-alpha and IL-6 (R&D Systems, Minneapolis, MN) on F1 sow’s plasma samples (D1L and D14L), colostrum (D1L) and milk (D14L). The sample were diluted according to the concentration expected to be detected for each analyte. For TFN-α, IL-6 and IL-10, the samples were used undiluted. For IgA, plasma was diluted 1:1,000, colostrum 1:30,000 and milk 1:2,000. For IgG, plasma was diluted 1:1,000,000, colostrum 1:1,000,000 and milk 1:2,000. For IgM, sow plasma was diluted 1:10,000, piglet plasma 1:50,000, colostrum 1:50,000 and milk 1:20,000. The plates were read on a microplate reader at 540 nm using a Multiskan Go (Thermo Fisher Scientific, EUA) evaluated by SkanIt Software 4.0 (Thermo Fisher Scientific, USA). The results were corrected for dilution factor.

### Analysis of eicosanoids by Liquid Chromatography Tandem-Mass Spectometry (LC-MS/MS)

As previously described by our colleagues [53], sows (batches A and B) and piglet (D13L) plasma samples were placed in a warm (37°C) water bath and stimulated for 20 min with thapsigargin (TAP) at the concentration 1.3 μM. The reactions were stopped by placing the samples in an ice bath. Samples were then centrifuged at 300 x g at ~4°C for 20 min and 300 μL of plasma was stored at -80°C until extraction and analysis. Dimethylsulfoxide (DMSO) served as a negative control. The analysis was carried out in collaboration with CEQIL–Center of Excellence in Quantification and Identification of Lipids, School of Pharmaceutical Sciences of Ribeirão Preto (FCFRP), University of São Paulo. TAP is calcium ionophore that we throw on the cell, so that the cell drops out whatever it produces as a lipid mediator. Other stimuli could be used, such as microorganisms and LPS (lipopolysaccharide). TAP was chosen due to it being the cleanest and safest method. DMSO is used as a solvent for TAP, and we tested the samples using only DMSO to verify if it could be harmful to the cell. The methodologies used in this project were developed by the CEQIL technical team and referenced below.

The methodology was used as previously described [54]. The lipids were purified by Solid Phase Extraction (SPE) using HPLC-grade solvents (Merck, Kenilworth, NJ, USA), added of deuterated internal standards (IS) were purchased from Cayman Chemical Co (Ann Arbor, MI, USA) and for the step columns Hypersep C18 (500 mg, 3 mL, Thermo Scientific-Bellefonte, PA, USA) were used. All the target analytes were quantified using high-resolution MRM (MRM^HR^), which are: LTB_4_, 6-trans-LTB_4_, 20-OH-LTB_4_, LTC_4_, LTD_4_, 11-trans-LTD_4_, LTE_4_, LXA_4_, RvD_1_, RvD_2_, TXB_2_, PGB_2_, PGE_2_, PGD_2_, 15-keto-PGE_2_, 20-OH-PGE_2_, PGD_2_, PGJ_2_,15-deoxi-δ-12,14-PGJ_2_, 6-keto-PGF_1_α, PGF_2_α, 19-OH-PGB_2_, PGG_2_, MaR-1, 5-HETE, AA, 5-oxo-ETE, 20-HETE, 5,6-DiHETE, 12-HETE, 8-HETE, 11-HETE, 12-oxo-ETE, 12-oxo-LTB_4_, 15-oxo-ETE, 11,12-DiHETrE, 14,15-DiHETrE, EPA, DHA, 15-HETE, 5,6-DiHETrE.

The liquid chromatography coupled with tandem mass spectrometry (LC-MS/MS) system (Nexera X2, Shimadzu-Kyoto, HO, Japan) and interfaced with a TripleTOF5600^+^ Mass Spectrometer (Sciex-Foster, CA, USA) which was conducted in the negative-ion mode, equipped with a Turbo-V IonSpray. Additional instrumental parameters were as follows: nebulizer gas (GS1), 50 psi; turbo-gas (GS2), 50 psi; curtain gas (CUR), 25 psi; electrospray voltage (ISVF), −4.0 kV; and turbo ion spray source temperature, 550°C. The mass range of the product ion experiments was from m/z 50 to 700 and the dwell time was 10 ms. The lipid species were identified using PeakView 2.1 (Sciex-Foster, CA, USA) and for quantitative analysis MultiQuant was used.

### Statistical analysis

All data were analyzed using SAS software (Version 9.4, SAS Institute Inc., Cary, NC). The experimental model followed a completely randomized design, the animal was considered as an experimental unit. Animals in pre-initial period were randomly assigned to 1 of 3 treatments. The normality of the residuals was verified by the Shapiro-Wilk test (PROC UNIVARIATE of SAS) and information with studentized residuals greater than +3 or less than −3 were excluded from the analysis. The homogeneity of variances was compared using the Levene test.

Variables with continuous distribution were analyzed using the MIXED procedure of SAS, and GLIMMIX when the non-normality of the residuals was founded. When the time factor was not present, the statistical model included: ‘treatment’ and ‘sex’ (when present) as a fixed effect, ‘animal’, ‘gestation period’, ‘lactation period’ and ‘residual’ as random effects. When the time factor was present, repeated measures in time were performed, in which the statistical model included: fixed effects of ‘treatment’, ‘time’, and ‘treatment-by-time interaction’. The condition of the umbilical cord was evaluated by the Chi-Square Test. Piglet weight at 1 and 14 days of life, the effect of piglet per sow, the weight of sows, order and time of birth, sex, were used as covariates, when appropriate.

The covariance structure for each parameter was determined based on the lowest value of the Akaike information criteria (AIC). Orthogonal contrasts were declared as: C1 (Control group vs. n-6+n-3) and C2 (n-6 vs. n-3). The data of hemolytic activity of the complement system was analyzed using ANOVA followed by Dunnett’s test for multiple comparisons. Effects were considered significant when P ≤ 0.05 and tendency towards significance at 0.05 < P < 0.10.

## Results

### Enhancing serum fatty acid profiles in female swine with biofortified milk

Our previous studies found that supplementing sows with milk enriched with n-3 and n-6 altered circulating fatty acid profiles of the sows prior to conception and on lactation day 1 [31, 32]. Here we report on the effect of treatment and study time point (D40G, D107, D1L and D21L) on fatty acid profiles of sow’s serum samples (Table 3; batch A). Compared with CON sows, levels of palmitoleic acid (P = 0.003) were elevated in sows biofortified with n-3 and n-6 milk during the gestation and lactation period. Furthermore, concentration of MUFA as elaidic (P = 0.035), palmitoleic (P = 0.003) and oleic acid (P = 0.040) were higher in the n-6 compared with n-3 group. Whereas the level of stearic acid, a SFA, was reduced (P = 0.004) in sows that received milk biofortified with PUFA n-3 compared with the n-6 group. Together these results led to a higher concentration of ΣMUFA for sows in n-6 group compared to sows in n-3 (P = 0.04).

**Table 3:** Effects of biofortified cow’s milk on the fatty acid profile (mg/mL) of sow serum during gestation and lactation.

Items^a^	Treatment^b^			*P-value* ^d^				
	CON (± SEM^c^)	n-6 (± SEM^c^)	n-3 (± SEM^c^)	Treatment	Time	Treat*Time	C1	C2
Myristic, C14:0	10.459 ± 0.10	9.489 ± 0.10	8.305 ± 0.10	0.324	<0.001	0.146	0.221	0.381
Palmitic, C16:0	199.160 ± 9.64	197.660 ± 9.48	184.640 ± 9.47	0.493	<0.001	0.170	0.497	0.336
Stearic, C18:0	138.212 ± 0.05	162.580 ± 0.05	134.328 ± 0.05	**0.008**	<0.001	0.147	0.201	**0.004**
Elaidic, C18:1t n-9	2.995 ± 0.07	3.497 ± 0.06	2.688 ± 0.07	0.102	<0.001	**0.007**	0.810	**0.035**
Palmitoleic, C16:1c9	10.334 ± 0.46	13.091 ± 0.42	11.136 ± 0.43	**<0.001**	<0.001	0.151	**0.003**	**0.003**
Oleic, C18:1c9	244.250 ± 11.34	267.420 ± 11.00	233.680 ± 11.00	0.106	<0.001	0.069	0.651	**0.040**
Linoleic, C18:2 n-6	330.580 ± 15.17	315.550 ± 14.83	298.290 ± 14.83	0.321	<0.001	0.454	0.207	0.415
Dihomo-γ-linolenic, C20:3 n-6	4.901 ± 0.45	4.975 ± 0.45	5.619 ± 0.45	0.448	<0.001	0.653	0.467	0.308
ARA, C20:4 n-6	106.910 ± 8.45	109.150 ± 8.45	93.225 ± 8.41	0.335	0.020	0.358	0.570	0.180
γ-Linolenic, C18:3 n-6	4.464 ± 0.05	4.527 ± 0.05	4.822 ± 0.05	0.598	<0.001	**0.008**	0.502	0.443
α-Linolenic, C18:3 n-3	6.511 ± 0.06	5.932 ± 0.05	6.362 ± 0.05	0.548	<0.001	0.091	0.476	0.425
EPA, C20:5 n-3	2.500 ± 0.06	2.881 ± 0.07	2.484 ± 0.06	0.391	<0.001	0.606	0.498	0.222
DHA, C22:6 n-3	2.644 ± 0.10	2.525 ± 0.10	1.900 ± 0.10	0.161	0.094	0.157	0.242	0.134
ΣSFA	354.640 ± 12.01	375.320 ± 13.24	345.600 ± 12.05	0.259	<0.001	0.058	0.695	0.109
ΣUSFA	717.600 ± 28.51	736.160 ± 27.66	659.930 ± 27.66	0.149	<0.001	0.168	0.577	0.063
SFA/USFA	0.490 ± 0.01	0.520 ± 0.01	0.510 ± 0.01	0.100	0.010	0.547	0.036	0.695
ΣMUFA	258.630 ± 12.02	284.260 ± 22.72	248.370 ± 11.72	0.104	<0.001	0.077	0.604	**0.040**
ΣPUFA	458.970 ± 19.20	451.900 ± 18.62	411.560 ± 18.62	0.177	<0.001	0.425	0.253	0.138
Σn-3	12.990 ± 0.59	13.000 ± 0.87	11.500 ± 0.63	0.124	<0.001	0.130	0.573	0.070
Σn-6	446.480 ± 18.69	435.700 ± 18.18	339.960 ± 18.18	0.189	<0.001	0.461	0.217	0.176
n-6/n-3	35.267 ± 0.03	34.075 ± 0.03	36.122 ± 0.03	0.436	<0.001	0.067	0.892	0.205
ARA/EPA	47.586 ± 3.47	37.536 ± 3.58	45.252 ± 3.67	0.118	<0.001	**0.045**	0.144	0.135

^a^ARA: arachidonic acid; EPA: eicosapentaenoic acid; DHA: docosahexaenoic acid; ΣSFA = Σ saturated fatty acids; ΣUSFA = Σ unsaturated fatty acids; SFA/USFA = Σ saturated/Σ unsaturated; ΣMUFA = Σ monounsaturated fatty acids; ΣPUFA = Σ polyunsaturated fatty acids; Σ n-3 = Σ omega-3 fatty acids; Σ n-6 = Σ omega-6 fatty acids; n-6/n-3 = Σ omega-6/Σ omega-3; ARA/EPA: arachidonic acid/eicosapentaenoic acid.

^b^Sows fed with a Control milk (CON), supplemented with cow’s milk biofortified with n-6 or n-3.

^c^SEM,standard error of the mean.

^d^C1, contrast Control vs n-3+n-6; C2, contrast n-3 vs n-6.

**Fig 3:**
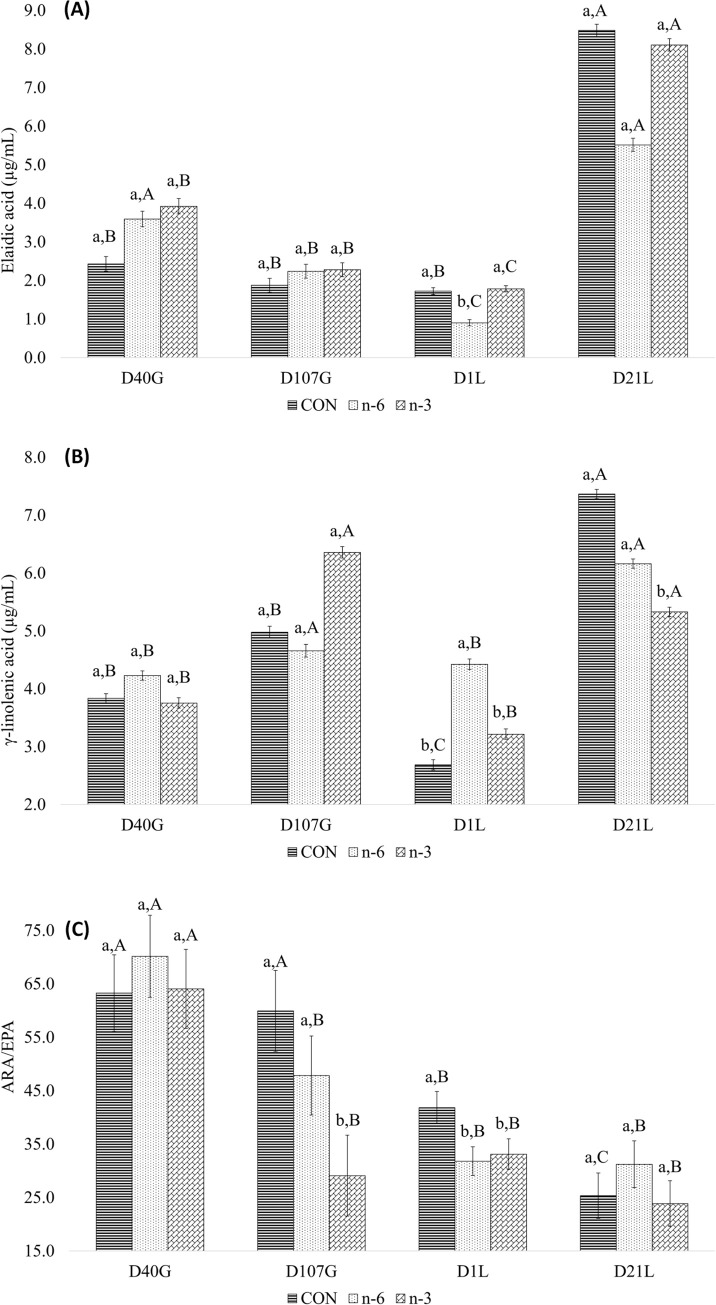
Effect of interaction treatments and time for elaidic (A), γ-linolenic (B) acid and ARA/EPA ratio (C) of sows. Average follow by the lowercase letter means the comparison among the treatments at one point in time. Average follow by the capital letter means the comparison of one treatment at different times. Means and P-values can be found in Tables 3–5 in **S1** File, respectively.

An interaction between treatment and time was observed for elaidic acid (P = 0.007), γ-linolenic acid (P = 0.008) and ARA/EPA ratio (P = 0.045) (Table 3). Elaidic level was affected by n-6 treatment at D1L, and by time. Between D40G and D1L, elaidic acid leves decreased to a greater extent in the n-6 and n-3 groups compared to CON group (Fig 3A and Table 3 in S1 File). Leves of γ-linolenic acid level in sow serum were also affected by treatments and time. The n-6 and n-3 groups lower levels of γ-linolenic acid on D40G and D1L, and higher levels on D107G and D21L (Fig 3B and Table 4 in S1 File). Biofortified milks supplementation lowered the ARA/EPA ratio in sows compared to the CON on D1L, and on D107G, n-3 milk supplementation resulted in a lower ratio compared to the CON and n-6. The ARA/EPA ratio did not vary across gestation and lactation time points in groups supplemented with biofortified milks, whereas in the CON group the ARA/EPA was lower during the lactation period (Fig 3C and Table 5 in S1 File).

### Biofortified milk with n-6 and n-3 affect backfat thickness in early gestation

**Fig 4:**
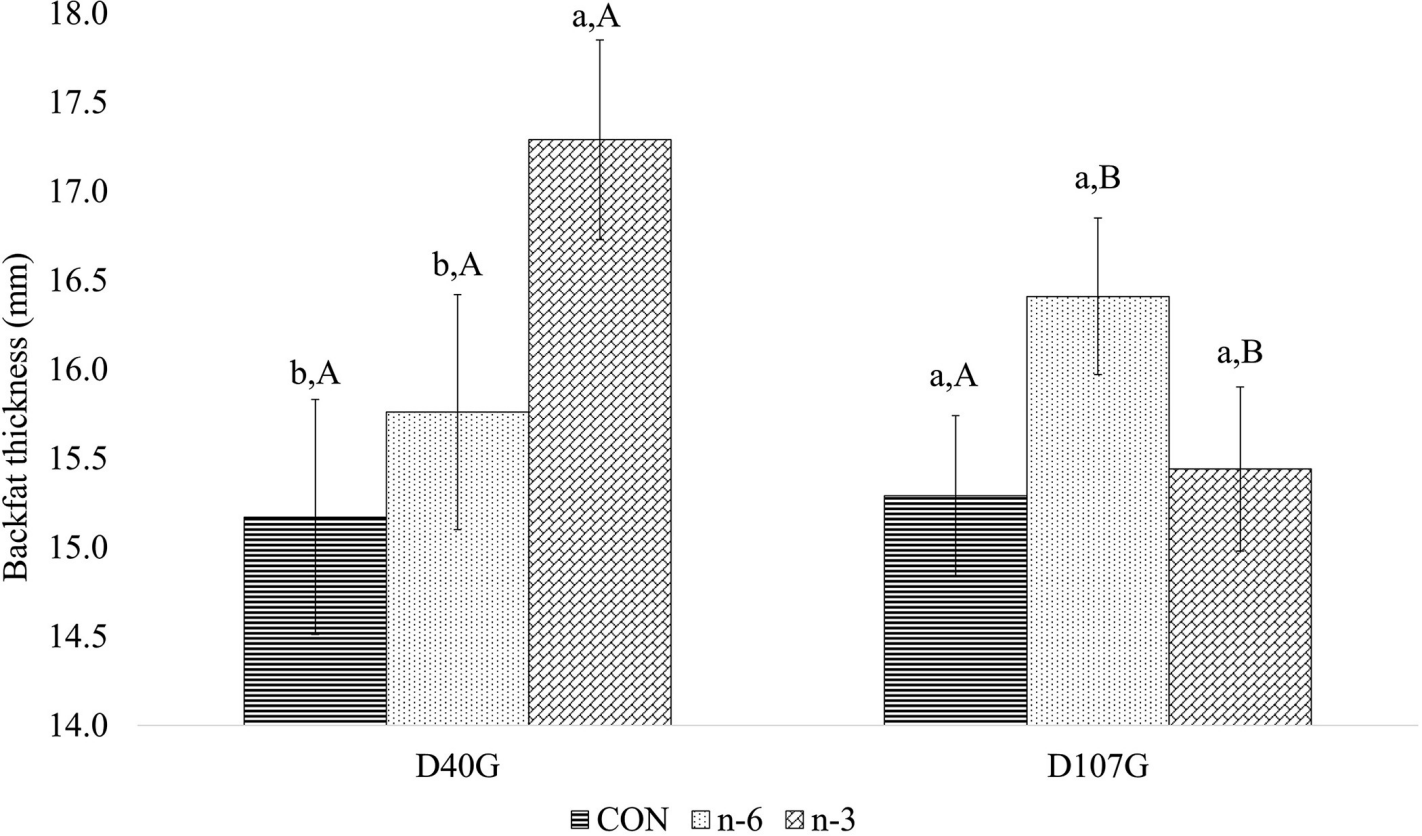
Effect of treatment and time on backfat thickness average of sows on early and late gestation. Average followed by the lowercase letter means the comparison among the treatments at one point in time. Average followed by the capital letter means the comparison of one treatment at different times. Means and P-values can be found in Table 6 in **S1** File.

We previously reported that supplementation of sows with biofortified milk had no effect on feed intake nor growth of animals from ~30 d of age to ~190 d [31]. Similarly, although sows gained weight across gestation, as indicated by a significant effect of time (P < 0.001), there was no effect of treatment on sow weight during gestation (Table 4). Treatment affected BF depth of sows in early gestation (D40G, P = 0.043), but not late gestation (D107G, P = 0.169). Post-hoc analysis indicated that animals fed biofortified milk had greater backfat than the CON group at D40G, (P = 0.014). Moreover, across gestation, the n-3 biofortified animals lost BF between D40G and D107G (- 0.26 mm) while n-6 biofortified milk supplementation increased BF depth (2.38 mm, P = 0.029). Interactions between treatment and time were observed in BF average (P = 0.010) (Table 4), wherein n-3 sow group had higher BF thickness at D40G but was not different from other groups at D107G (Fig 4). BF thickness did not vary across study time points in the CON group, whereas sows in the n-3 group reduced thickness and n-6 group increased BF thickness (Fig 4).

**Table 4:** Effect of supplementation of milk biofortified with PUFA n-6 and n-3 on the backfat thickness of sows from D40 to D107 of gestation.

	Treatment^b^			*P-value* ^d^				
Item^a^	CON (± SEM^c^)	n-6 (± SEM^c^)	n-3 (± SEM^c^)	Treatment	Time	Treat*Time	C1	C2
Body weight average, kg	185.32 ± 3.55	181.06 ± 3.98	188.38 ± 3.050	0.389	**<0.001**	0.270	0.196	0.433
Weight D40G, kg	162.44 ± 3.35	162.50 ± 3.35	166.44 ± 3.16	0.610	-	-	0.325	0.990
Weight D107G, kg	208.57 ± 4.92	202.79 ± 4.34	205.67 ± 4.34	0.682	-	-	0.998	0.388
Weight difference, D107G - D40G, kg	46.35 ± 1.08	43.91 ± 1.08	41.20 ± 1.08	0.562	-	-	0.360	0.562
Backfat thickness average, mm	15.08 ± 0.42	16.07 ± 0.37	16.36 ± 0.34	0.086	0.920	**0.010**	0.091	0.102
Backfat thickness D40G, mm	14.30 ± 0.66	14.73 ± 0.61	16.42 ± 0.54	**0.043**	-	-	**0.014**	0.640
Backfat thickness D107G, mm	16.09 ± 0.37	17.01 ± 0.39	16.10 ± 0.39	0.169	-	-	0.346	0.096
Backfat thickness difference, D107G - D40G, mm	1.44 ± 0.74	2.38 ± 0.78	-0.26 ± 0.83	0.084	-	-	0.686	**0.029**

^a^D40G: 40 days of gestation; D107G: 107 days of gestation.

^b^Sows fed with a Control milk (CON), supplemented with cow’s milk biofortified with n-3 or n-6.

^c^SEM, standard error of the mean.

^d^C1, contrast Control vs n-6+n-3; C2, contrast n-6 vs n-3.

### Impact of biofortified cow’s milk on serum biochemistry in female swine

**Fig 5:**
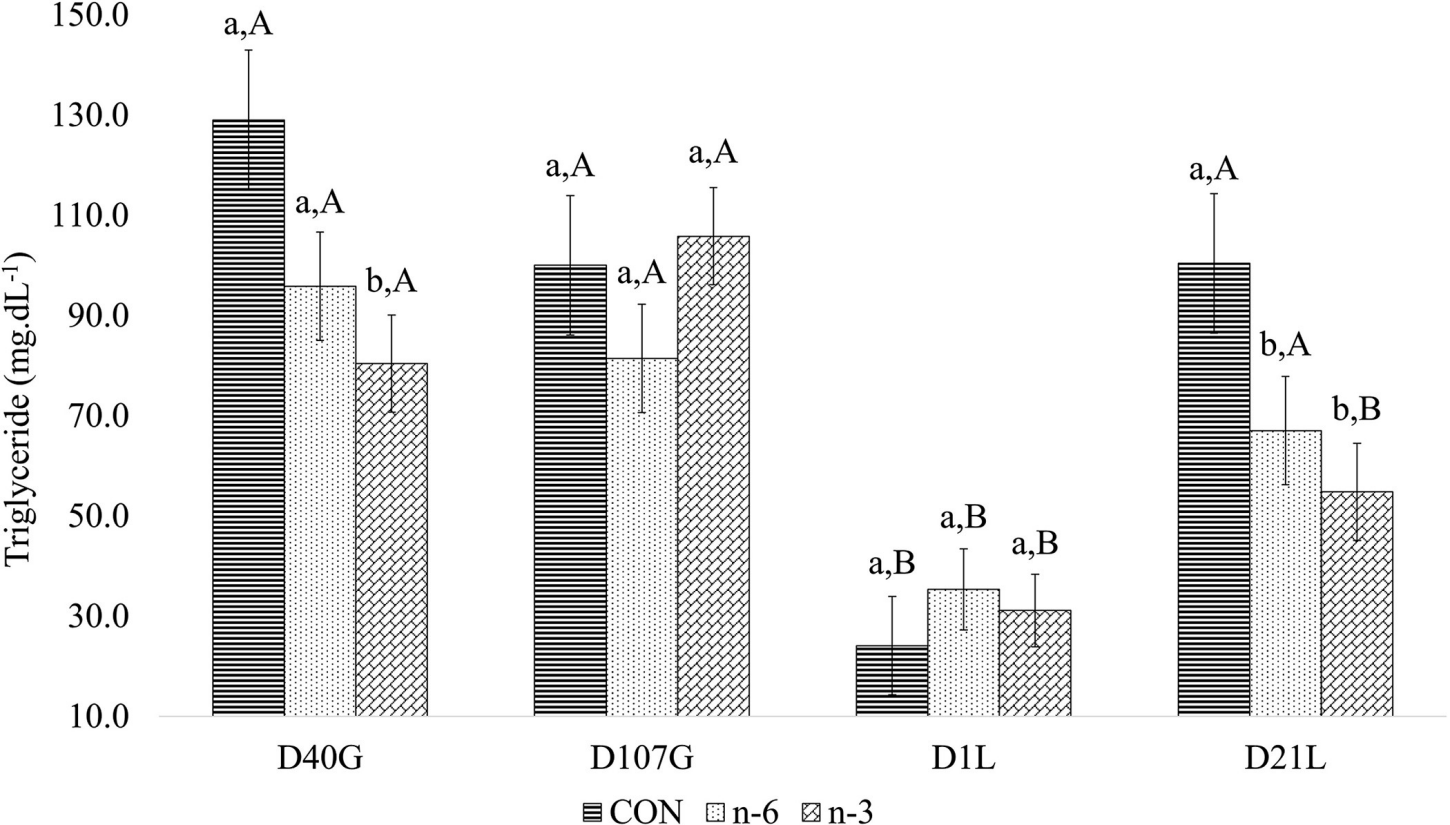
Effect between treatments and time for triglyceride of swine female. Average followed by the lowercase letter means the comparison among the treatments at one point in time. Average followed by the capital letter means the comparison of one treatment at different times. Means and P-values can be found in Table 7 in **S1** File.

Circulating levels of urea, total protein, total cholesterol, HDL, LDL, VLDL, glucose, and TG in sows varied by gestation and lactation day (batch A). Treatment affected VLDL and TG levels. Sows supplemented with n-3 milk had higher VLDL concentration than those supplemented with n-6 milk. N-3 and n-6 groups had lower levels of TG than CON group (Table 5). Treatment by time interactions were found for circulating concentrations of TG (Fig 5). Levels of TG were lower on D1L compared to both gestation time points and D21L. Within these time periods treatment differences varied (Fig 5). On D40G and D21L, TG was lower in n-3 group. Across the days, CON and n-6 were higher in gestation period and D21L and lower in D1L. Biofortified milks reduced the TG levels on D21L.

**Table 5:** Effects of biofortified cow’s milk on the biochemical parameters of blood of swine female during gestation and lactation.

	Treatments^a^			*P-value* ^c^				
Parameters	CON (± SEM^b^)	n-6 (± SEM^b^)	n-3 (± SEM^b^)	Treatment	Time	Treat*Time	C1	C2
Urea, mg/dL	24.36 ± 0.06	24.89 ± 0.06	28.25 ± 0.06	0.189	0.0097	0.753	0.263	0.149
Total protein, g/dL	7.31 ± 1.75	8.12 ± 1.75	8.46 ± 1.75	0.589	<0.001	0.132	0.940	0.309
Total cholesterol, mg/dL	66.85 ± 2.82	62.86 ± 2.75	63.78 ± 2.92	0.578	<0.001	0.725	0.314	0.820
HDL, mg/dL	27.71 ± 1.77	26.54 ± 1.77	25.84 ± 1.77	0.723	<0.001	0.634	0.451	0.761
LDL, mg/dL	26.97 ± 2.79	24.15 ± 2.79	22.77 ± 2.79	0.332	<0.001	0.357	0.163	0.601
VLDL, mg/dL	6.46 ± 2.99	4.26 ± 2.99	7.84 ± 2.99	**0.030**	<0.001	0.061	0.500	**0.011**
Glucose, mg/dL	101.08 ± 2.74	97.20 ± 2.74	104.31 ± 2.74	0.313	<0.001	0.083	0.920	0.131
Triglycerides, mg/dL	88.38 ± 6.51	69.88 ± 5.10	68.03 ± 4.57	**0.039**	<0.001	**0.040**	**0.012**	0.788

^a^Sows fed with a Control milk (CON), supplemented with cow’s milk biofortified with n-6 or n-3.

^b^SEM, standard error of the mean.

^c^C1, contrast Control vs n-6+n-3; C2, contrast n-6 vs n-3.

### Colostrum production and intake

Treatment had no effect on the quantity of colostrum produced by sows nor on colostrum intake of piglets (P > 0.05, Table 8 in S1 File).

### Viability: Apgar and umbilical cord after supplementation with biofortified milk

**Fig 6:**
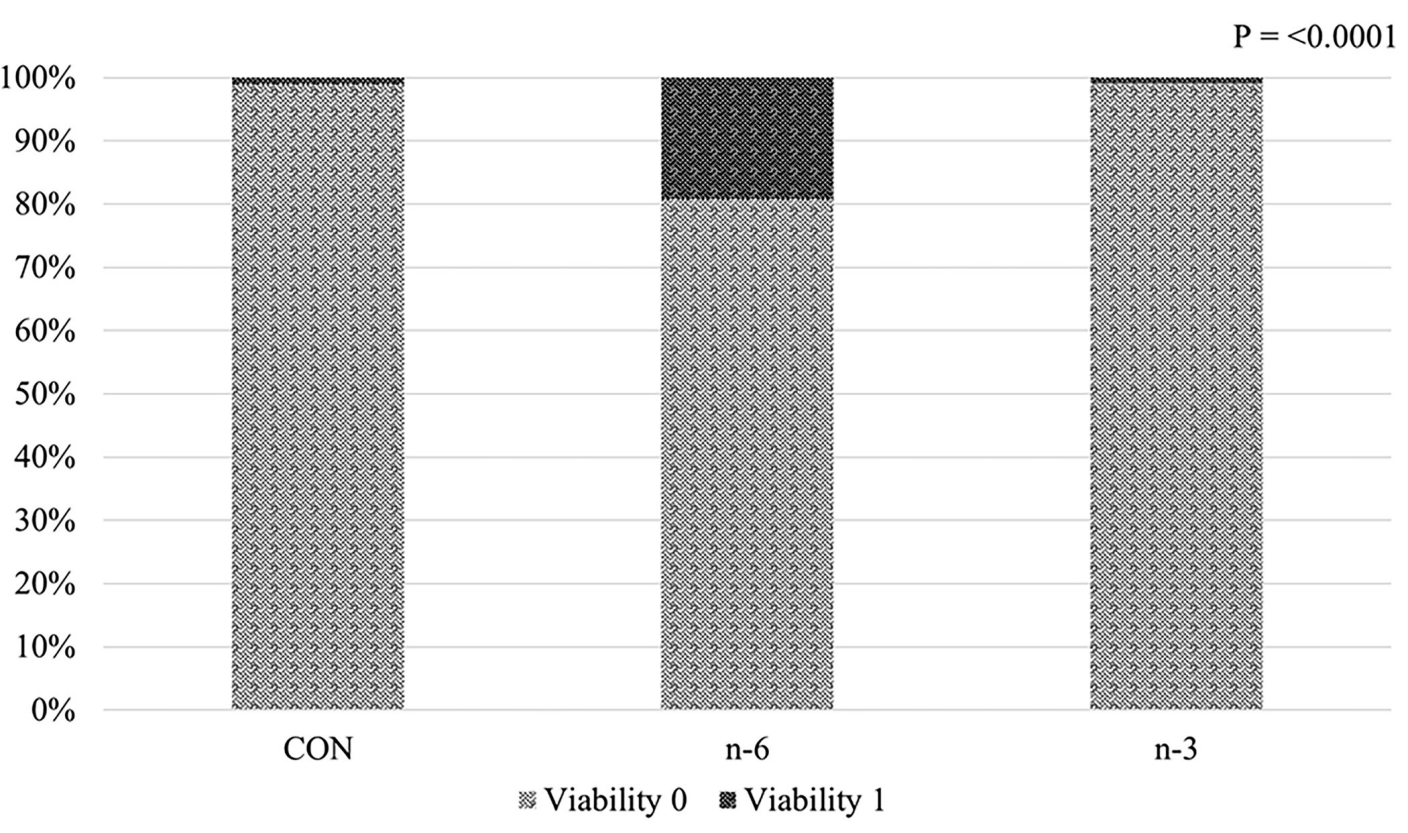
Effects of supplementing biofortified milk of pregnant sows on proportion of newborn piglets’ viability 0 or 1 classified according to the Apgar score.

**Fig 7:**
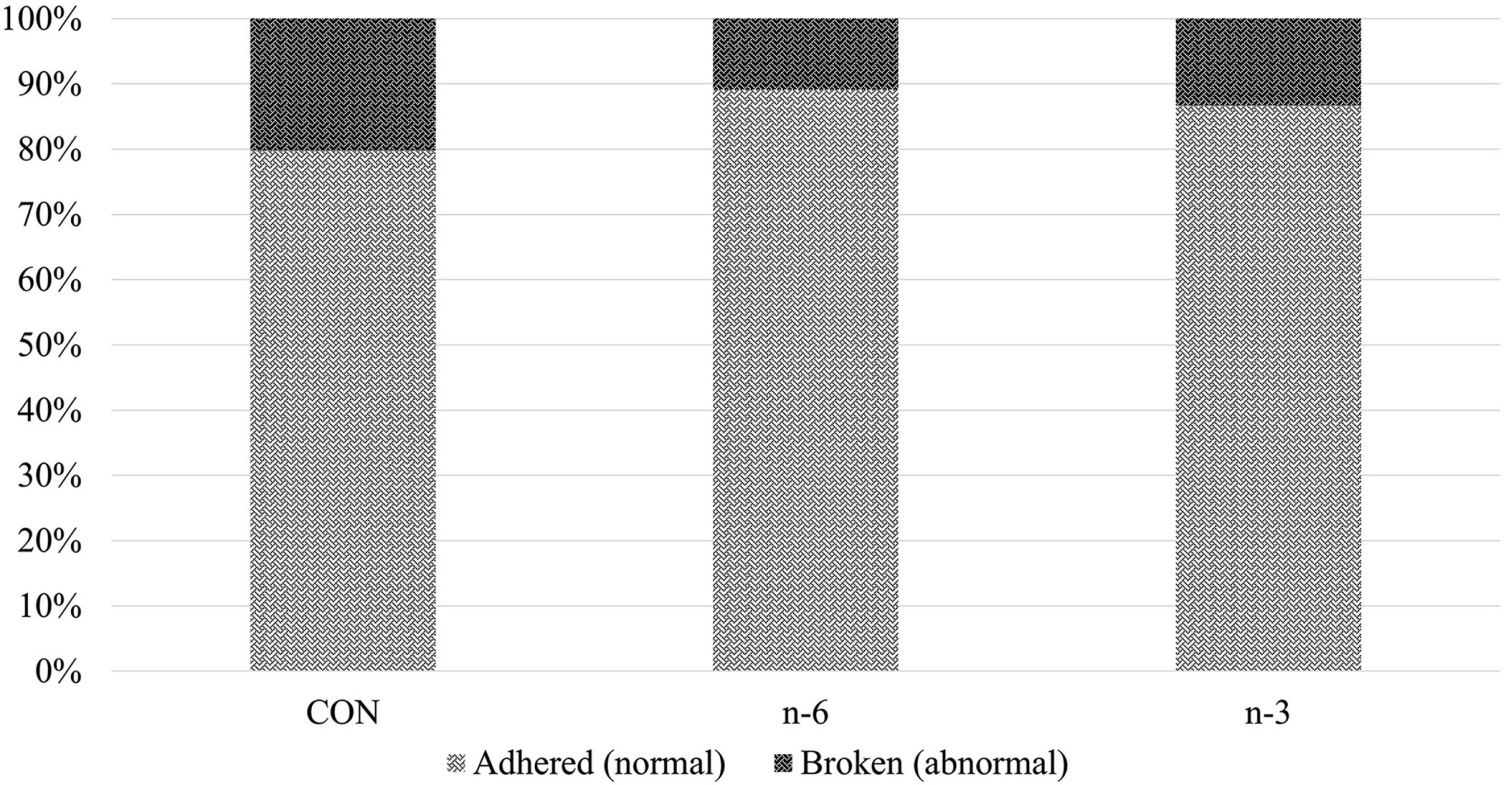
Condition of the umbilical cord of newborn piglets classified according to the treatment CON, n-6 or n-3.

The Apgar score (batch A) was used to evaluate the effect of maternal diet on piglet vitality right after birth, with the higher score indicating a better state of health. Piglets born with vitality score of 1, representing low survival, had a frequency of 1.01%, 19.28% and 0.95%, for CON, n-6 and n-3, respectively (Fig 6). There was no difference (P > 0.05) in percentage of offspring with adhered or ruptured umbilical cords (Fig 7).

### Biofortified milk increases the total number of embryos at 28 days of gestation

Relative to control supplemented group, the total number of embryos were increased in both the n-6 and n-3 groups (P = 0.048). N-6 and n-3 groups also tended to have more viable embryos (P = 0.060) compared with CON treatment (Table 6).

**Table 6:** Survival of porcine embryos after sow’ supplementation with control, omega-3 and omega-6 milk at 28 days of gestation.

	Treatment^b^			*P-value* ^d^		
Item^a^	CON (± SEM^c^)	n-6 (± SEM^c^)	n-3 ± (SEM^c^)	Treatment	C1	C2
Number of embryo’s, n						
Viable	15.00 ± 1.27	17.57 ± 1.11	16.67 ± 0.94	0.099	**0.060**	0.185
Unviable	0.38 ± 0.18	0.75 ± 0.41	0.33 ± 0.24	0.297	0.738	0.154
Total	15.43 ± 1.32	18.43 ± 1.07	17.00 ± 0.94	**0.060**	**0.048**	0.101
Corpora lutea, n	18.75 ± 1.06	20.25 ± 1.05	18.44 ± 1.30	0.301	0.712	0.132
FR^2^, %	80.78 ± 6.61	83.38 ± 5.11	95.15 ± 7.40	0.266	0.321	0.206
AFR^3^, %	78.70 ± 6.71	79.90 ± 5.12	93.43 ± 7.63	0.233	0.360	0.156
Pregnant uterus, kg	4.850 ± 0.544	4.749 ± 0.518	5.293 ± 0.473	0.695	0.916	0.408

^a^FR: Fertilization ratio = (Number of embryo’s total/Corpora lutea) x 100; AFR: Adjusted Fertilization ratio = (Number of embryo’s viable/Corpora lutea) x 100.

^b^Sows fed with a Control milk (CON), supplemented with cow’s milk biofortified with n-6 or n-3.

^c^SEM: standard error of the mean.

^d^C1, contrast Control vs n-6+n-3; C2, contrast n-6 vs n-3.

### Determination of the microstructure of *Longissimus dorsi*

Dietary supplementation had no effect on area, minimum, medium, and maximum diameters of muscle fibers and adipocytes in *Longissimus dorsi* of sows nor their offspring (Tables 9 and 10 in S1 File). However, piglet sex significantly impacted (P < 0.05) the area, minimum and medium diameter of adipocytes with males having greater measures than females.

### Relative expression levels of genes related to regulating lipid homeostasis, metabolism, and appetite

The relative expression of several genes that control lipolysis and lipogenesis was measured in liver tissue collected from sows (batch B) and piglets (batch A), also genes responsible for appetite control were measured in arcuate nucleus of the hypothalamus of sows. There was no effect of n-3 or n-6 supplementation on hepatic expression of ACC, FADS2, FAS, SCD, PPAR-α, nor SREBP-1 genes in sows (P > 0.05) (Table 11 in S1 File). Hepatic expression of SREBP-1 in offspring was 75.8% greater in the n-3 group compared to n-6 group (P = 0.033) (Table 7). Also, the SCD gene was affected by sex (P = 0.038), with higher relative expression in males (1.241 ± 0.182) than females (0.944 ± 0.170). There was no effect (P > 0.05) of treatment on expression of ARH genes in sows (Table 12 in S1 File).

**Table 7:** Liver expression levels of genes involved in lipid metabolism in piglets 14-day-old from treated sows–batch A.

Item	Treatment^b^			*P-value* ^d^				
	TC (± SEM^c^)	n-6 (± SEM^c^)	n-3 (± SEM^c^)	Treat	Sex	Treat*Sex	C1	C2
ACC	1.054 ± 0.095	0.830 ± 0.103	0.879 ± 0.125	0.286	0.449	0.594	0.137	0.768
FADS2	1.589 ± 0.381	0.847 ± 0.284	1.275 ± 0.217	0.295	0.248	0.305	0.234	0.254
FAS	0.980 ± 0.215	0.946 ± 0.242	1.572 ± 0.548	0.665	0.442	0.752	0.564	0.457
SCD	1.310 ± 0.278	0.671 ± 0.131	1.297 ± 0.249	0.226	**0.038**	0.825	0.555	0.113
PPAR-α	0.983 ± 0.124	0.945 ± 0.129	0.922 ± 0.140	0.946	0.319	0.599	0.754	0.909
SREBP-1	0.951 ± 0.163	0.909 ± 0.162	1.596 ± 0.240	0.074	0.489	0.376	0.189	**0.033**

^a^Sows fed with a Control milk (CON), supplemented with cow’s milk biofortified with n-6 or n-3.

^b^SEM: standard error of the mean.

^c^C1, contrast Control vs n-6+n-3; C2, contrast n-6 vs n-3.

### Increased hemolytic activity of complement system in sows supplemented with biofortified milk and their piglets

**Fig 8:**
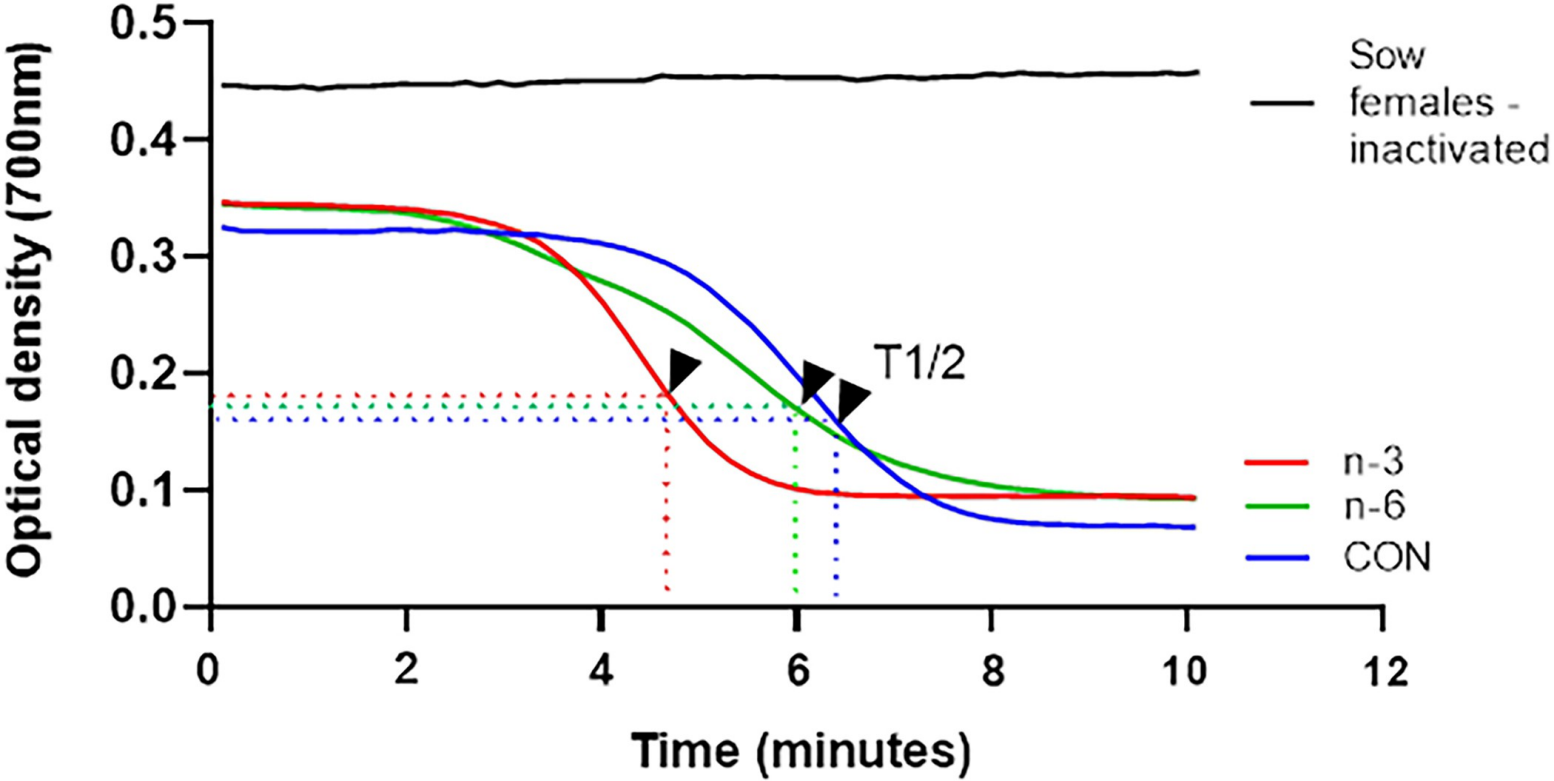
Hemolytic activity of the alternative pathway of the complement system—batch A by kinetic assay. Serum samples from the animals in the control, n-6 and n-3 groups were tested without dilution. A: lytic curves of the mean of each experimental group and the negative control of complement system activity (inactivated sow serum pool).

The results of hemolytic activity of complement system from batch A are represented in Fig 8. In this assessment, the T1/2 values were calculated from undiluted samples, per animal in each group: control, n-6, and n-3. Black arrows indicate the value of T1/2 in minutes for each group.

**Fig 9:**
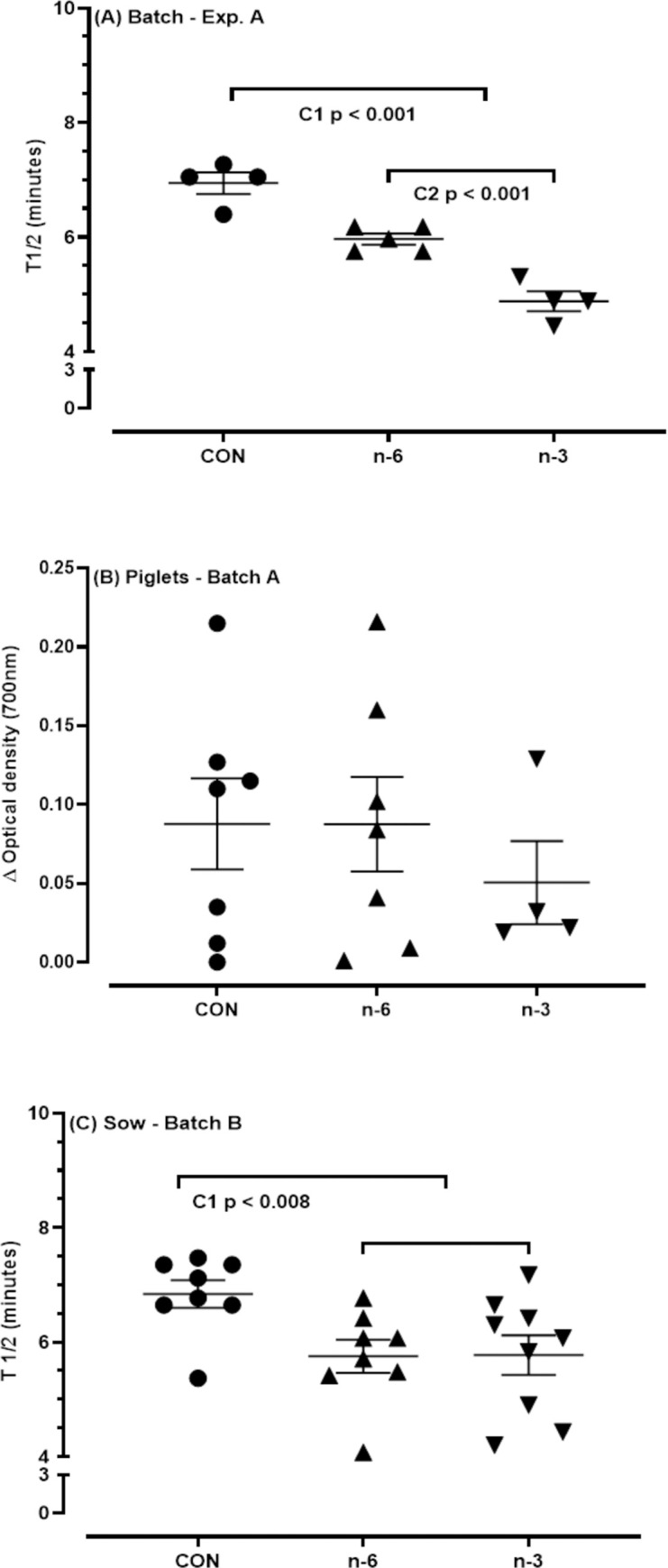
T1/2 values (minutes) determined as the hemolytic activity of the alternative pathway of the complement system of the serum from batch A, sows (A) and piglets (B), and batch B, sows (C). Sow fed with a Control milk (CON), supplemented with cow’s milk biofortified with n-6 or n-3. T1/2 values in minutes for samples from the respective experimental groups analyzed in duplicates. T1/2, time required for complement to reduce 50% of the initial optical density of the rabbit erythrocytes suspension. The T1/2 values for each sample in the groups correspond to the average of duplicate trials. Serum samples from piglets T1/2 values were based on the variation between initial and final optical density (ΔOD). The bars indicate the means ± standard error of the mean. C1, contrast Control vs n-6+n-3; C2, contrast n-6 vs. n-3. Means and P-values can be found in Table 13 in **S1** File.

Hemolytic activity of sow complement system was higher in n-6 and n-3 biofortified milk groups than CON (batches A and B, Fig 9A, 9C). Moreover, the n-3 group had a more rapid response in their organism compared to n-6 group (Fig 9A). However, the treatments did not influence the complement in their piglets (Fig 9B).

### Immunoglobulin and interleukin concentrations in colostrum, milk, plasma of sows, and piglets

Circulating levels of IgA (P < 0.001), IgG (P = 0.004), and IgM (P = 0.001) in sows significantly varied across study time point (Table 8). Treatment affected plasma IgG, with levels significantly lower (P = 0.011) in sows supplemented with biofortified milk than CON group (Table 8). Plasma TNF-α levels were significantly lower (P = 0.042) in CON sows than n-6 and n-3 sows, and higher in n-3 sows relative to the n-6 group (P = 0.001; Table 8). A significant treatment by time interaction was found for circulating levels of IgM (P = 0.011) and TNF-α (P = 0.009) observed for sows (Table 8).

**Table 8:** Concentration of the immunoglobulins and interleukins in plasma of sows as the response to supplementation during the gestation and lactation period with biofortified cow’s milk.

	Treatment^a^			*P-value* ^c^				
Item	CON (± SEM^b^)	n-6 (± SEM^b^)	n-3 (± SEM^b^)	Treatment	Time	Treat*Time	C1	C2
IgA, mg.mL^-1^	4.346 ± 0.781	3.911 ± 0.731	4.161 ± 0.781	0.852	**<0.001**	0.599	0.583	0.860
IgG, mg.mL^-1^	203.730 ± 13.647	149.600 ± 13.525	155.49 ± 13.647	**0.037**	**0.004**	0.401	**0.011**	0.891
IgM, mg.mL^-1^	20.111 ± 3.451	20.751 ± 3.261	19.155 ± 3.451	0.839	**0.001**	**0.011**	0.983	0.555
TNF-α, pg.mL^-1^	29.030 ± 2.845	31.639 ± 2.506	37.265 ± 2.908	0.261	0.120	**0.009**	**0.042**	**0.001**
IL-6, pg.mL^-1^	n.d.	n.d.	n.d.	n.d.	n.d.	n.d.	n.d.	n.d.
IL-10, pg.mL^-1^	n.d.	n.d.	n.d.	n.d.	n.d.	n.d.	n.d.	n.d.

^a^Sows fed with a Control milk (CON), supplemented with cow’s milk biofortified with n-6 or n-3.

^b^SEM: standard error of the mean.

^c^C1, contrast Control vs n-6+n-3; C2, contrast n-6 vs n-3.

n.d: not detectable. The values were not estimated due to missing, as a consequence of the limitations of the method to measure the concentration in the sample base on the standard curve.

**Fig 10:**
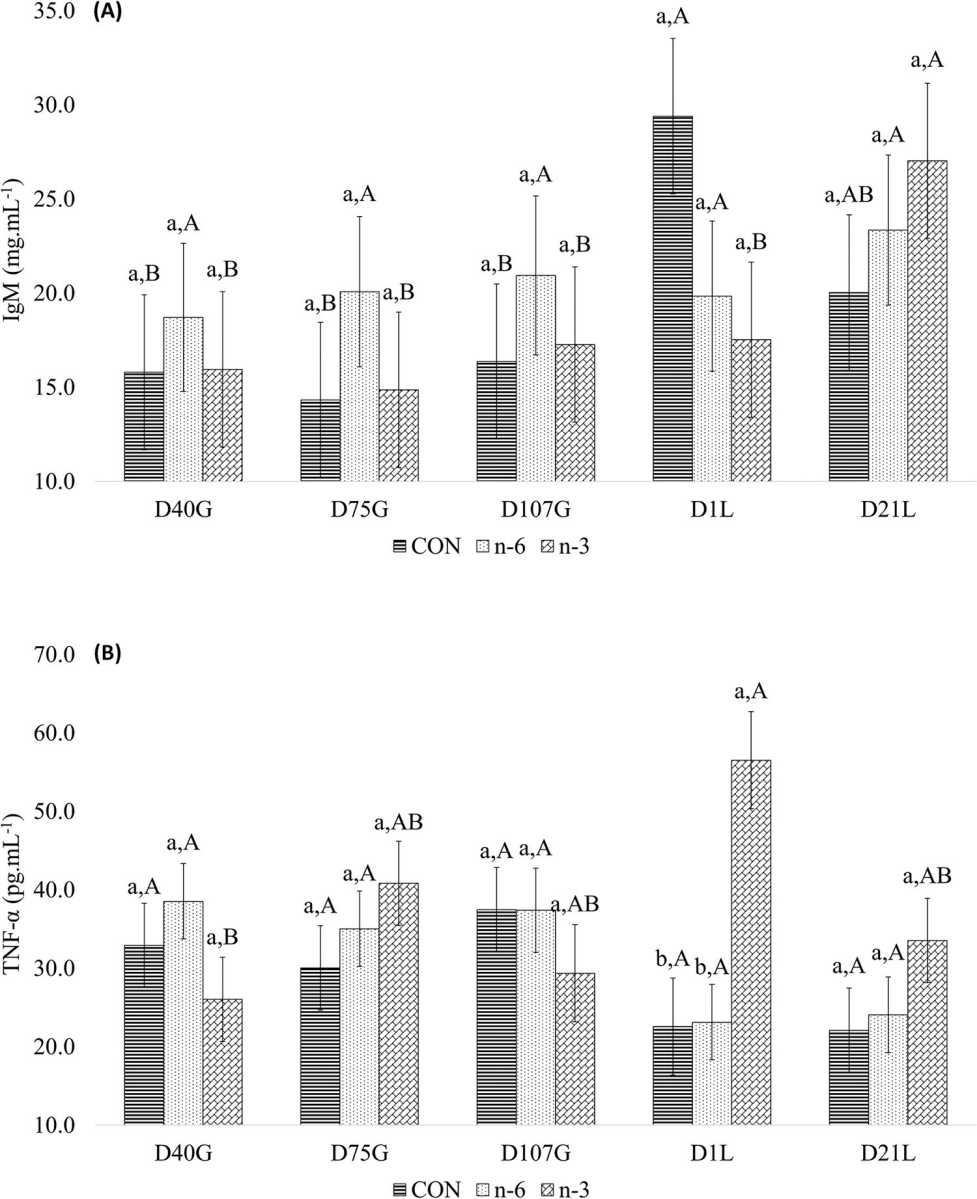
Increased IgM (A) and TNF-α (B) levels in sow’s plasma. Average followed by the lowercase letter means the comparison among the treatments at one point in time. Average followed by the capital letter means the comparison of one treatment at different times. Means and P-values can be found in Tables 14 and 15 in **S1** File, respectively.

On D1L plasma circulating levels of IgM in sows were higher in CON group than biofortified milk groups. On D21L IgM level was greatest for n-3 group compared to CON and n-6. Circulating levels of IgM did not vary across study time points in the sows of n-6 group (Fig 10A). In the n-3 group, plasma circulating TNF-α levels varied by time and relative to other treatments in sows increasing on D1L (56.5314 pg.mL^-1^) (Fig 10B).

IgG levels in colostrum (D1L) of n-6 and n-3 groups were 51% lower than CON group (from 137.601 mg.mL^-1^ to 67.862 mg.mL^-1^ average) (Table 9). The n-3 group tended (P = 0.063) to have higher IL-6 level in sow colostrum (Table 9) compared to n-6 biofortified milk.

**Table 9:** Total immunoglobulin and interleukin concentration in the colostrum of sows.

	Treatment^a^			*P-value* ^d^		
Item	CON (± SEM^b^)	n-6 (± SEM^b^)	n-3 (± SEM^b^)	Treatment	C1	C2
IgA, mg.mL^-1^	16.460 ± 4.577	15.920 ± 2.895	20.182 ± 2.895	0.573	0.758	0.325
IgG, mg.mL^-1^	137.601 ± 1.599	57.862 ± 1.520	77.862 ± 1.731	**0.050**	**0.022**	0.378
IgM, mg.mL^-1^	9.294 ± 0.218	7.497 ± 0.165	6.723 ± 0.165	0.535	0.312	0.668
TNF-α, pg.mL^-1^	n.d.	n.d.	n.d.	n.d.	n.d.	n.d.
IL-6, pg.mL^-1^	35.810 ± 47.211	26.593 ± 29.859	115.960 ± 29.859	0.139	0.510	**0.063**
IL-10, pg.mL^-1^	n.d.	n.d.	n.d.	n.d.	n.d.	n.d.

^a^Sows fed with a Control milk (CON), supplemented with cow’s milk biofortified with n-6 or n-3.

^b^SEM: standard error of the mean.

^c^C1, contrast Control vs n-6+n-3; C2, contrast n-6 vs n-3.

n.d: not detectable. The values were not estimated due to missing, as a consequence of the limitations of the method to measure the concentration in the sample base on the standard curve.

IgG levels tended to be lower in mature milk of both n-6 and n-3 groups (0,079 and 0,277 mg.mL^-1^, respectively) than CON (Table 10).

**Table 10:** Total immunoglobulin and interleukin concentration in the milk of sows on day 14 of lactation.

	Treatment^a^			*P-value* ^d^		
Item	CON (± SEM^b^)	n-6 (± SEM^b^)	n-3 (± SEM^b^)	Treatment	C1	C2
IgA, mg.mL^-1^	n.d.	n.d.	n.d.	n.d.	n.d.	n.d.
IgG, mg.mL^-1^	0.781 ± 0.069	0.702 ± 0.077	0.504 ± 0.069	**0.043**	**0.064**	**0.082**
IgM, mg.mL^-1^	1.905 ± 0.445	1.732 ± 0.445	1.607 ± 0.445	0.929	0.722	0.905
TNF-α, pg.mL^-1^	n.d.	n.d.	n.d.	n.d.	n.d.	n.d.
IL-6, pg.mL^-1^	n.d.	n.d.	n.d.	n.d.	n.d.	n.d.
IL-10, pg.mL^-1^	n.d.	n.d.	n.d.	n.d.	n.d.	n.d.

^a^Sows fed with a Control milk (CON), supplemented with cow’s milk biofortified with n-6 or n-3.

^b^SEM: standard error of the mean.

^c^C1, contrast Control vs n-6+n-3; C2, contrast n-6 vs n-3.

n.d: not detectable. The values were not estimated due to missing, as a consequence of the limitations of the method to measure the concentration in the sample base on the standard curve.

Mean levels of IgA, IgM and TNF-α in piglet plasma were (P < 0.05) higher in the n-6 and n-3 offspring than those of CON group (Table 11), whereas and IL-10 was lower. Time also impacted piglet plasma IgA, IgG and IgM levels (P < 0.001) (Table 11). Levels of IgA in piglet serum were 79.2% higher (P = 0.001) in n-6 group (3.360 mg.mL^-1^) compared to n-3 group (1.875 mg.mL^-1^). IL-10 were 135.5% higher in the n-3 group compared to the n-6 (10.657 *vs*. 4.526 pg.mL^-1^) (Table 11).

**Table 11:** Concentration of the immunoglobulins and interleukins in plasma of piglets at day 1 and 14 of lactation.

	Treatment^b^			*P-value* ^d^				
Item^a^	CON (± SEM^c^)	n-6 (± SEM^c^)	n-3 (± SEM^c^)	Treatment	Time	Treat*Time	C1	C2
IgA, mg.mL^-1^	1.131 ± 0.342	3.360 ± 0.361	1.875 ± 0.362	**<0.001**	**<0.001**	0.629	**0.011**	**0.001**
IgG, mg.mL^-1^	75.418 ± 14.845	83.742 ± 29.391	46.983 ± 15.127	**0.024**	**<0.001**	**0.002**	0.211	0.073
IgM, mg.mL^-1^	4.898 ± 0.742	6.283 ± 1.181	4.445 ± 0.757	0.058	**<0.001**	**<0.001**	**0.047**	0.272
TNF-α, pg.mL^-1^	45.643 ± 18.278	86.595 ± 18.287	88.594 ± 18.278	**0.007**	-	-	**0.002**	0.781
IL-6, pg.mL^-1^	n.d.	n.d.	n.d.	n.d.	n.d.	n.d.	n.d.	n.d.
IL-10, pg.mL^-1^	14.816 ± 3.126	4.526 ± 3.297	10.657 ± 2.699	**0.021**	0.796	0.126	**0.027**	**0.044**

^a^The variable TNF-α had only the day 14 of lactation included on the statistical analysis as a consequence of the limitations of the method to measure the concentration in the sample based on the standard curve.

^b^Sows fed with a Control milk (CON), supplemented with cow’s milk biofortified with n-6 or n-3.

^c^SEM: standard error of the mean.

^d^C1, contrast Control vs n-6+n-3; C2, contrast n-6 vs n-3.

n.d: not detectable. The values were not estimated due to missing, as a consequence of the limitations of the method to measure the concentration in the sample base on the standard curve.

**Fig 11:**
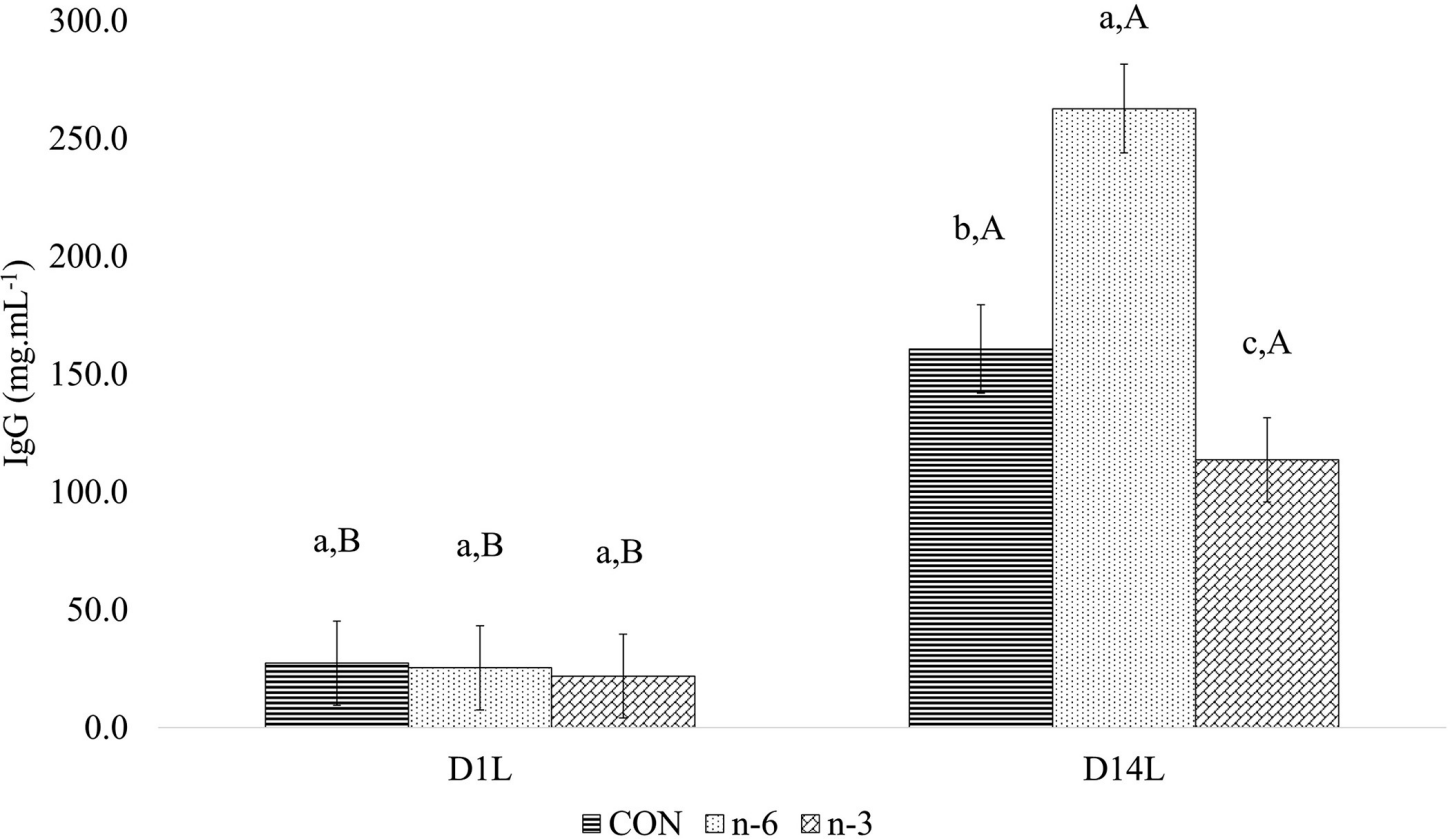
Effect between treatments and time in plasma for IgG of piglets. Average followed by the lowercase letter means the comparison among the treatments at one point in time. Average followed by the capital letter means the comparison of one treatment at different times. Means and P-values can be found in Table 16 in **S1** File.

**Fig 12:**
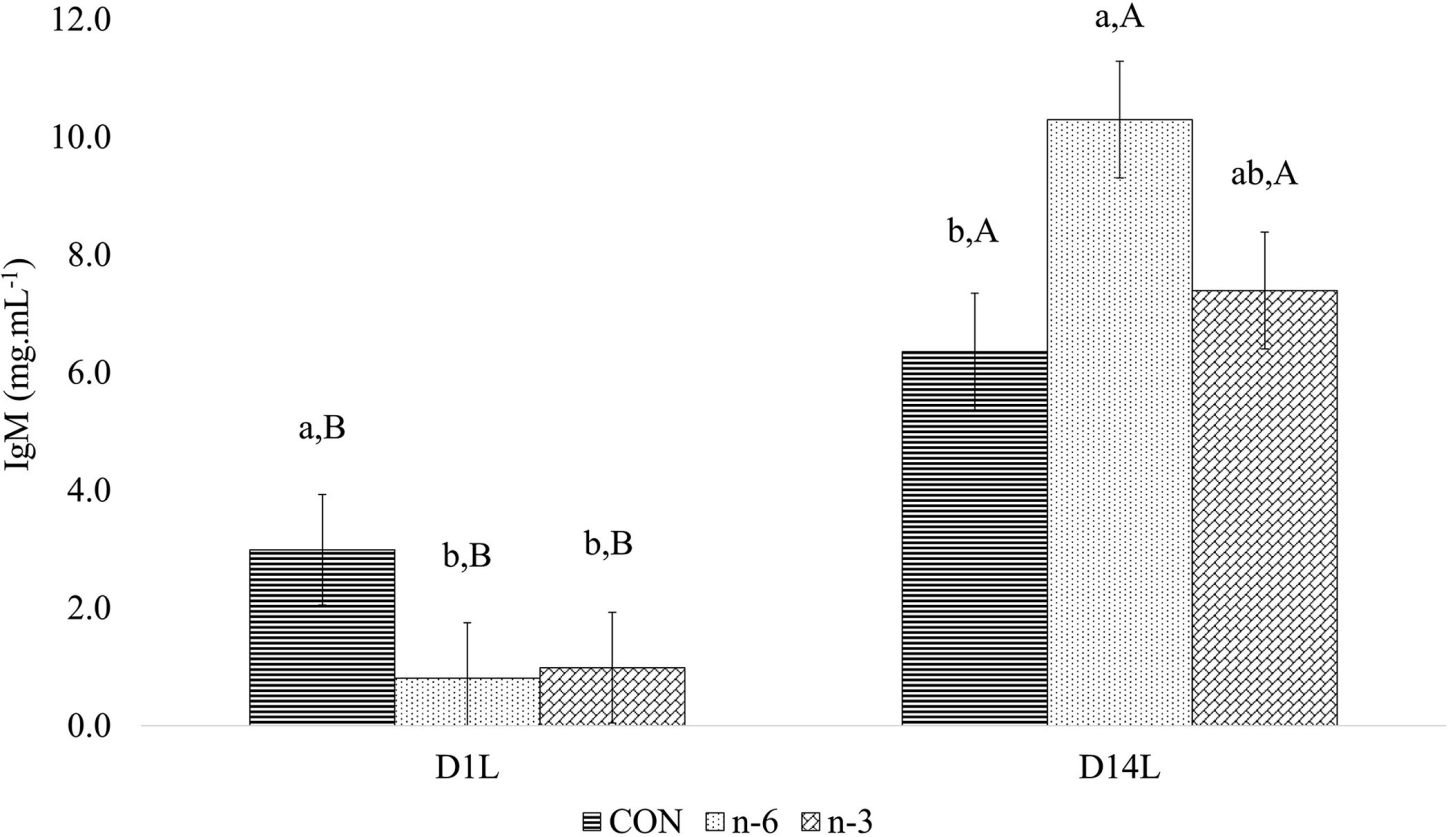
Effect between treatments and time in plasma for IgM of piglets. Average followed by the lowercase letter means the comparison among the treatments at one point in time. Average followed by the capital letter means the comparison of one treatment at different times. Means and P-values can be found in Table 17 in **S1** File.

There was a treatment and time interaction for circulating IgG (P = 0.002) and IgM (P < 0.001) (Table 11) levels in piglets. The mean IgG (Fig 11) and IgM (Fig 12) concentrations increased (P < 0.05) from D1L to D14L for all treatments. However, on D14L the mean of IgG level was significantly (P < 0.05) lower in piglets of n-3 biofortified sow by 29.27% and 56.73%, than CON (160.69 mg.mL^-1^) and n-6 (262.69 mg.mL^-1^) groups, respectively (Fig 11).

When the mean IgM concentration was compared between the control and supplemented groups, it was noted that mean IgM was significantly (P < 0.05) lower on D1L for n-6+n-3 than CON group, and 39.21% and 61.92% higher (P < 0.05) on D14L for n-6 group than n-3 and CON group, respectively (Fig 12).

### Analysis of eicosanoid plasma profiles in sows and piglets after PUFA supplementation

All metabolites outlined in the materials and methods section underwent testing, however, only LTB4, PGF2α, and TXB2 were quantifiable. There were no differences (P > 0.05) between treatment groups or contrasts applied for LTB4, PGF2α or TXB2. The stimulation with TAP or DMSO had a significant effect (P = < 0.0001) for TXB2 on both sows and piglets of batch A (Tables 18 and 19 in S1 File), and sows of batch B (Table 20 in S1 File).

## Discussion

Long-term supplementation of female swine with milk biofortified with n-6 and n-3 PUFA led to changes in immune and metabolic status of sows were coincident with differences in embryo viability, weight of piglets at birth, colostrum and milk fatty acid profiles, postnatal growth rate, and related to differences in their offspring, including circulating fatty acid profiles, immune status and hepatic expression of lipogenic genes. Both n-3 and n-6 milk enhanced total embryos, increased activity of complement system, decreased hepatic expression of SREBP-1 and increased SCD expression in piglets, however, neonates of n-6 supplemented sows showed a tendency for lower viability-vigor at birth as indicated by Apgar score.

Metabolic and behavioral adaptions are initiated in females with the onset of pregnancy that promote the increase of fat and protein stores during the first two-thirds of pregnancy. Females then enter a catabolic state in which they mobilize protein and fat stores to support the nutrient and energetic demands of the growing fetus and milk production in late gestation and early lactation [55, 56]. During the interval of D40G and D107G in our study, the sow transitioned from an anabolic to catabolic state. N-3 and n-6 PUFA supplementation through cow’s milk increased (P = 0.014) backfat thickness by 8.92% D40G than CON. N-3 group mobilized more backfat (P = 0.029) from D40 to D107 of gestation, while the n-6 group, and CON had an increment of 15.5% and 12.5% respectively. These differences suggest that n-3 biofortified milk better supported maternal adaptations to reproductive states reflected by the higher average body weight and daily gain for the piglets from sows that received biofortified milk [32]. However, differences in backfat mobilization were not associated with improved colostrum production, as there was no difference (P < 0.05) in estimated amount of colostrum produced among the groups. This finding is not consistent with previous reports of changes in backfat thickness during late gestation associating with colostrum production [57].

In the present study, the biofortified milk promoted the stability of the sows’ body condition (BF between 14 and 17 mm) without affecting the total piglets born, born alive, stillborn or mummified [32], despite others reporting that thinner sows produce smaller litters and fewer born alive [58].

Current healthy eating guidelines recommend low-fat dairy, as whole milk is ~60% SFA, which increase serum cholesterol and triglycerides and lower relative abundance of PUFA [59]. Studies of humans found a balance between SFAs and PUFAs, plays a crucial role in determining TG circulating levels. TG concentration had negative associations with C20:3 n-6, C20:4 n-6, C20:5 n-3, and C22:5 n-3, and positive associations with palmitic acid and C18:3 n-3. The surface response analysis indicated that the impact of palmitic acid on triglyceride levels varied based on the chemical structure of PUFAs [60].

Biofortified cow’s milk used as a treatment in the present study demonstrated excellent nutritional quality in the assessed lipid fraction through atherogenicity index, thrombogenicity index, and the ratio of hypocholesterolemic to hypercholesterolemic fatty acids [27], as it has lower SFA/USFA ratio, increased MUFA and Σn-3. Alterations in fatty acid profiles of sows fed n-3 biofortified milk resulted in lower circulating TG levels on D40G and D21L. Palmitoleic acid (C16:1c9), which is attributed to reduce hypertriglyceridemia in mouse model [61] were also higher in sows supplemented with n-3 and n-6 biofortified milk. FA serum has a rule in metabolism regulating proteins and lipolytic enzyme activity [62].

Our previous studies found that the fat, protein, lactose or solids-not-fat of colostrum and milk was not affected by treatment with biofortified milk [32]. Here we report that feeding sows biofortified milk modified fatty acids in colostrum and milk, as well as in piglet’s serum. These changes were related to increase growth performance of piglets in litters of sows receiving biofortified milk. Since the macronutrient content of milk was not different, the differences in growth may be due effects of fatty acids on metabolic pathways that regulate, which in turn may improve outcomes in various aspects as conception rates, embryogenesis, blood flow, antioxidant activity, appetite, and other cellular development processes. PUFA can cross the placenta into the fetal circulation [63].

Apgar scoring system, employed to evaluate neonatal vitality by assessing non-invasive physiological traits of newborns within one minute after birth [43], lacks scientific evidence regarding how the n-6/n-3 ratio impacts on the vitality of the piglets. The intriguing aspect of these findings is that the n-6 group displayed a tendency for poorer viability relative to the n-3 group, and neonates took a longer time to initiate suckling after birth [32]. These findings indicate that the offspring’ Apgar is more negatively influenced by mother’s supplementation with cow’s milk n-6 PUFA biofortified.

Feeding sows biofortified milk tended to improve the viability of embryos in 14,1% (P = 0.06) and significantly increased of the total number of embryos in 14,8% (P = 0.048). This finding suggests that maternal intake of n-3 and n-6 PUFA may enhance periconception and post-conception environments by promoting the development of high-quality follicles, optimizing ova size, and regulating key reproductive processes at the molecular level. Derivatives of long-chain polyunsaturated fatty acids (LC-PUFA) play crucial roles in signaling associated with maternal recognition of pregnancy. Pig oocytes exhibit elevated levels of LC-PUFAs, particularly n-6 fatty acids like LA and ARA, which enhance the rate of embryo growth [64–66]. The incorporation of these PUFAs into oocytes significantly impacts follicle development, resulting in the production of larger ova that are more likely to be successfully ovulated and fertilized post-insemination. Furthermore, the incorporation of these PUFAs can regulate the expression of genes responsible for prostaglandin and sex steroid information [67]. Other studies have reported that sows supplemented with fish oil as a source of n-3 PUFA experienced an increase litter size in subsequent parities [64]. However, our previous study [32] showed that the total born in batch A did not differ among the treatments which used vegetable oil to biofortify milk.

In exploring the molecular aspects, PPAR-α and SREBP are a ligand-activated transcriptional factors and regulate the expression of FADS2 (fatty acid desaturase 2), which in turn regulate the activity of delta-6 desaturase (D6D) enzyme, which is one of two rate limiting enzymes that convert the PUFA. LC-PUFA and their derivatives actively mediate metabolic pathways and cellular signaling resulting in the suppression of SREBP-1 and upregulation of PPAR-α, and in turn decreasing the expression of lipogenic genes such as ACC, FAS and SCD [68–72].

In the present study, piglets from sows fed n-3 biofortified milk exhibited a significantly up-regulated expression of SREBP-1 gene. SREBP-1 stimulates FA and TG synthesis (*de novo* synthesis). This leads to oxidative stress and, consequently, n-3 PUFA depletion [73].

Up-regulation of SREBP-1 could be attributed to the reduction of ARA in sows’ milk, as PUFA has the potential to decrease its expression [73], however, several factors actively influence hepatic mRNA expressions of SREBP-1 [74]. Current findings remain insufficient to fully elucidate SREBP-1 expression and warrant further research as initiating lipogenesis in the early post-partum is important for piglets in their mediation of thermogenesis.

Male piglets had higher SCD expression in the liver and larger adipocytes in *L*. *dorsi*. Studies of mice and cattle also found body composition of neonates varies by sex and may be attributed to the hormonal and fatty acids composition in their bodies [75–77]. Once SCD plays a crucial role in metabolic process, is reasonable suggest the possibility of a link between SCD expression and *L*. *dorsi* adipocytes and its potential as molecular target to modulate porcine fatness [78].

Supplementing sows with biofortified milk modulated the innate and adaptive systems. Sows fed biofortified milk had a higher activation capacity of the complement system, with even greater activation in n-3 group compared to n-6 biofortified animals. However, because these are specific points measured, it is not possible to evaluate the duration of this condition, which if elongated, can bring damage to the organism.

The treatments’ impact indicated an increase in the hemolytic activity of the complement system, and it carries significant implications for immunology and health. The complement system plays a crucial role in conditions such as insulin resistance, atherosclerosis, and obesity, with adipose tissue synthesizing key proteins that regulate it [79]. High-fat diets, particularly those rich in saturated fats (SFA) and n-6 polyunsaturated fats (PUFA), are identified as potential contributors to inflammation and the subsequent activation of the complement system [79–81]. Overall, the current study demonstrates that altering the FA profile of biofortifying milk can alter the activity of the complement system. However, the actual benefits hinge on the clinical context and the specific disease in question.

There was no effect of sow supplementation on complement system activity in piglets. Whether lack of a difference is due to no effect of maternal diets on offspring complement system activity, or that differences may be manifested at a later stage, when the immune system is matured, remains to be tested.

A decreased amount of antibodies and pro-inflammatory interleukins were expected in the sows and piglets of the n-3 group at this study, once the PUFA can modestly reduce the stimulation of B cells, through the suppression of antigen presentation and a decrease in antibody production, as well as a reduction in pro-inflammatory cytokines [82, 83]. However, there is a disparity in the literature that reports the opposite effect of the stimulation of n-3 PUFA [73, 83, 84].

The survival of piglets relies heavily on the acquisition of passive immunity through colostrum and milk from sows. In this study, we shown that milk biofortified with PUFA increased the levels of IgA, IgM, and TNF-α, while decreasing IL-10 in the plasma of piglets compared to the CON group. Additionally, n-3 PUFA decreased IgA and increased IL-10 compared to n-6 PUFA, potentially due to its effect on B cells, enhancing anti-inflammatory capacity. Considering the observed interactions, the highest concentrations of IgG and IgM at D14L, compared to D1L, suggested the development of active immunity in piglets. Our results indicate that the effect of treatments on progeny occurred through the lactation phase for IgG, regardless of placental levels. However, for IgM, differences were evident even before the first sucking, with a higher concentration in n-6 group at D14L. Notably, IL-6 was not detected in mature milk, contrasting with its presence in colostrum.

Since colostrum from sows in the CON group showed higher IgG concentration compared to n-6 and n-3, a higher concentration of IgG was expected in the plasma of piglets of CON at D14L. However, the n-6 group showed the highest IgG concentration, followed by CON and n-3 group. Currently, the mechanism through which n-6 and n-3 PUFA alter the concentrations of IgG, IgA, and IgM remains unclear, also the relationship between the n-6/n-3 ratio and the changes in immunoglobulin concentrations in plasma is not yet well understood.

Maternal supplementation with n-3 and n-6 PUFA increased the pro-inflammatory interleukin TNF-α and decreased the anti-inflammatory IL-10 compared to CON group. However, the increase of IL-10 in piglet’s plasma indicates that sows receiving biofortified milk with linseed oil (n-3 group) might improve the anti-inflammatory capability in suckling piglets compared to n-6 group, which can be attributed to the impact of cytokines, given their influence on T cells and production by different T helpers.

Quite unexpectedly, biofortified milk n-3 PUFA elevated TNF-α levels in the sow’s plasma compared to n-6 group, as well as both compared to CON. This finding contradicts the observations in literature [85], which upon supplementation of miniature pigs with flaxseed oil can reduce TNF-α in blood serum. In addition, evidence leads to believe that maybe the inclusion of low-ratio n-6/n-3 PUFA supplementation in the diet significantly reduces TNF-α levels in sick individuals, whereas it may not be replicated in healthy individuals [86]. IgM is well-known for being the first antibody produced in immune response, effectively activating the complement system and controlling bloodstream infections [87].

Indeed, the n-6 and n-3 treatments significantly lowered IgG levels, which serves as the primary neutralizer for toxins found in tissues. Earlier gestation period is highly sensitive, involving the establishment of gestation. At this crucial stage, biofortified milk n-3 PUFA mitigated the level of this antibody in the blood. This outcome is noteworthy, considering that the organism should recognize the fetuses to maintain the pregnancy. IgG had a higher concentration in colostrum (137.6 mg.mL^-1^, P = 0.022) and in CON sow’s milk (0.781 mg.mL^-1^, P = 0.064), which can be explained by the higher amount in plasma for the CON group (203.730 mg.mL^-1^, P = 0.011), as the transport occurs from serum into colostrum and milk [88].

In this study, biofortified milk demonstrated its ability to modulate the adaptive immune system. For a better understanding, future studies need explore supplementation in challenge organismic situations. Additionally, when observing the significant results overall, for first contrast (Control vs n-6+n-3), it is necessary to acknowledge that the effects may arise not only from biofortified milk with n-6 and n-3 PUFA, but also from increased levels of USFA, MUFA and Σn-3 PUFA, less SFA and lower SFA/USFA and n-6/n-3 ratio. Furthermore, the contrast between n-6 and n-3 allows attributing the differences to milk from cows that received linseed oil (n-3 group) with higher Σn-3 and lower n-6/n-3 ratio, as well as milk from cows that received soybean oil in the diet (n-6 group) with higher Σn-6 concentration, a higher n-6/n-3 ratio, and a tendency (P = 0.097) for higher cholesterol concentration. It is important to note that different species, varied dosages, variable durations of interventions, and diverse sources of lipids in the literature contribute to a range of information that can be challenging to compare. However, these factors, in combination, may contribute to the observed results, without considering the interaction of these lipid fractions with other compounds present in the body or from the diet.

## Conclusion

Together findings indicated that the consumption of biofortified milk with altered n-6/n-3 ratios or rich in n-3 PUFA reduced backfat thickness and circulating triglycerides, improved the blood FA profile, reducing MUFA and lower ARA/EPA ratio. In colostrum the profile became richer in n-3 PUFA, changing the n-6/n-3 ratio, as well as in milk this ratio was also modified. The worst viability of piglets was observed in group n-6. Biofortified milk showed immunomodulatory potential by making the response time of the complement system faster, and altering immunoglobulins and interleukins in the colostrum, milk and blood of sows and their litter. Furthermore, the expression of genes involved in lipid metabolism needs to be better studied, not for their expression but for their action and release, suggesting a regulatory potential, which can be further investigated as benefits for health issues, for sows or their offspring. The effect of biofortified milk also benefited the total number of embryos, showing the effect on the reproductive system. While some results agreed with the hypothesis, others diverged from the expected effects, however, these results provide valuable insights into the effects of biofortified milk consumption. Further studies are warranted to elucidate the underlying mechanisms and determine the long-term health implications of these dietary interventions, with potential applications in both animal production and human health promotion.

## Supporting information

S1 File Supplementary tables. (PDF)

S2 File Supplementary information on each premix used in feed. (PDF)

S3 File Supplementary information–dataset. (XLSX)
