# Supplementary material for: Feeding sows milk biofortified with n-6 and n-3 modulates immune status of sows and drives positive transgenerational effects
Source: PLoS One. 2024 Aug 27;19(8):e0306707. doi: 10.1371/journal.pone.0306707 (PMC11349115; doi:10.1371/journal.pone.0306707)
Supplement: S1 File — (PDF) [file pone.0306707.s001.pdf]

Feeding sows milk biofortified with n-6 and n-3 modulates immune status of sows and drives positive transgenerational effects

Authors: Leriana Garcia Reis[1*,#a], Vera Letticie de Azevedo Ruiz[2&], Simone Maria Massami Kitamura[3], André Furugen Cesar Andrade[4], Fernando de Oliveira Bussiman[5], Mirele Daiana Poleti[2], Juliano Coelho da Silveira[2], Heidge Fukumasu[2], Lúcia Helena Faccioli[6], Cleni Mara Marzocchi-Machado[6], Ricardo de Francisco Strefezzi[2], Edna Neves Garcia[3], Theresa Casey[1], Arlindo Saran Netto[3,#b].

Short Tittle: Biofortified Milk's Immune Modulation in Sows and Transgenerational Benefits with n-6 and n-3

[1]Department of Animal Science, Purdue University, West Lafayette, Indiana, USA.

[2]Department of Veterinary Medicine, School of Animal Science and Food Engineering, University of São Paulo, Pirassununga, SP, Brazil.

[3]Department of Animal Science, School of Animal Science and Food Engineering, University of São Paulo

[4]Department of Animal Reproduction, School of Animal Science and Food Engineering, University of São Paulo

[5]Department of Animal and Dairy Science, University of Georgia, Athens Georgia , USA.

[6]Department of Clinical Analyses, Toxicology and Food Sciences, School of Pharmaceutical Sciences of Ribeirão Preto, University of São Paulo, Ribeirão Preto, SP, Brazil.

[#a]Current address: Department of Animal Science, Purdue University, West Lafayette, Indiana 47907, USA

[#b]Current address: Department of Animal Science, School of Animal Science and Food Engineering, University of São Paulo, Rua Duque de Caxias Norte, 225, 13635-900, Pirassununga, SP, Brazil

*Corresponding author:

Email: lgarciar@purdue.edu/lerianagarcia@outlook.com (L.G.R.)

Key words: Essential fatty acids. Immune modulation. Maternal nutrition. Piglets. Swine reproductive outcomes.

Table 1. Gene names and primer sequences of liver

**Gene abbreviation[a]**	**Gene name**	**Primer sequences (5’-3’)**	**GenBank sequence**	**References**
ACC	Acetyl-CoA Carboxylase	F: ATGTTTCGGCAGTCCCTGAT<br>R: TGTGGACCAGCTGACCTTGA	NM_001114269.1	Chen et al. (2017)
SREBP-1	Sterol Regulatory Element-Binding Protein 1	F: CTACCACAAGCTGCACCAG<br>R: GGGAGACTGGTCTTGACTCG	NM_214157.1	Li et al. (2016)
SCD	Stearoyl-CoA Desaturase	F: TTGATCCCCACCTGCAAGAT<br>R: CGTGTTGGCAATGATCAGGA	NM_213781.1	Cirera et al. (2020)
PPAR-α	Peroxisome Proliferator Activated Receptor Alpha	F: TTTCCCTCTTTGTGGCTGCT<br>R: GGGGTGGTTGGTCTGCAAG	NM_001044526.1	Mentzel et al. (2018)
FADS2	Fatty acid desaturase 2	F: ACATGAACCTGTTCGAGAGC<br>R: TGATGAACTTGTGGACGATG	NM_001171750.1	Meadus, et al. (2011)
FAS	Fatty Acid Synthase	F: GTGGGCTACAGCATGATAGG<br>R: GAATTGCAGCGAGGAGTTAG	NM_001099930.1	Meadus, et al. (2011)
HPRT1	Hypoxanthine Phosphoribosyltransferase 1	F: AAGGACCCCTCGAAGTGTTG<br>R: CACAAACATGATTCAAGTCCCTG	NM_001032376.2	Svobodová, Bílek and Knoll (2008)
ACTB	β-Actin	F: TCTGGCACCACACCTTCT<br>R: TGATCTGGGTCATCTTCTCAC	NM_021086047.1	Jiao, et al. (2020)

[a]Housekeeping genes: HPRT1 and ACTB.

Table 2. Gene names and primer sequences of ARH

**Gene abbreviation[a]**	**Gene name**	**Primer sequences (5’-3’)**	**GenBank sequence**	**References**
CART	Cocaine and Amphetamine-Related Transcript	F: CCGCCCTGCTGCTGCTGCTAC<br>R: AGGGACTTGGCCATACTTCTTCTC	NM_001099925.1	Óvilo et al. (2010)
INSR	Insulin Receptor	F: AAACGCCAGGGACATCGTCAAGG<br>R: CCGCAGGGAACGCAGGTAACTCT	XM_021083940.1	Óvilo et al. (2014)
LEPR	Leptin Receptor	F: GAAAAACACCGGAATGATGC<br>R: AAAAGAAGAGGGCCAAATGTC	NM_001024587.1	Óvilo et al. (2014)
NPY	Neuropeptide Y	F: TCGGCGTTGAGACATTACATCA<br>R: GTCTCGGGACTAGATCGTTTTCC	NM_001256367.1	Óvilo et al. (2010)
HPRT1	Hypoxanthine Phosphoribosyltransferase 1	F: AAGGACCCCTCGAAGTGTTG<br>R: CACAAACATGATTCAAGTCCCTG	NM_001032376.2	Svobodová, Bílek and Knoll (2008)
ACTB	β-Actin	F: TCTGGCACCACACCTTCT<br>R: TGATCTGGGTCATCTTCTCAC	NM_021086047.1	Jiao, et al. (2020)

[a]Housekeeping genes: HPRT1 and ACTB.

Table 3. Concentration of elaidic acid (µg/ mL) at 40 and 107 days of gestation and at 1 and 21 days of lactation of sows

	**Gestation**		**Lactation**	
**Treatment**	**D40G[a] (± SEM[b])**	**D107G[a] (± SEM[b])**	**D1L[a] (± SEM[b])**	**D21L[a] (± SEM[b])**
CON	$2.430 \pm 0.19^{a,B}$	$1.875 \pm 0.18^{a,B}$	$1.725 \pm 0.09^{a,B}$	$8.482 \pm 0.16^{a,A}$
n-6	$3.593 \pm 0.20^{a,A}$	$2.237 \pm 0.18^{a,B}$	$0.903 \pm 0.08^{b,C}$	$5.514 \pm 0.17^{a,A}$
n-3	$3.922 \pm 0.20^{a,B}$	$2.279 \pm 0.18^{a,B}$	$1.782 \pm 0.08^{a,C}$	$8.105 \pm 0.16^{a,A}$

Average on the same line followed by the same capital letter, do not differ. Averages in the same column followed by the same lowercase letter, do not differ.

[a]D40G: 40 days of gestation; D107G: 107 days of gestation; D1L: first day of lactation; D21L: 21 days of lactation.

[b]SEM, standard error of the mean.

Table 4. Concentration of γ-linolenic acid (µg/mL) at 40 and 107 days of gestation and at 1 and 21 days of lactation of sows

	**Gestation**		**Lactation**	
**Treatment**	**D40G[a] (± SEM[b])**	**D107G[a] (± SEM[b])**	**D1L[a] (± SEM[b])**	**D21L[a] (± SEM[b])**
CON	$3.835 \pm 0.08^{a,B}$	$4.979 \pm 0.10^{a,B}$	$2.686 \pm 0.09^{b,C}$	$7.365 \pm 0.08^{a,A}$
n-6	$4.228 \pm 0.08^{a,B}$	$4.658 \pm 0.11^{a,A}$	$4.425 \pm 0.09^{a,B}$	$6.162 \pm 0.08^{a,A}$
n-3	$3.754 \pm 0.09^{a,B}$	$6.358 \pm 0.10^{a,A}$	$3.216 \pm 0.09^{b,B}$	$5.329 \pm 0.08^{b,A}$

Average on the same line followed by the same capital letter, do not differ. Averages in the same column followed by the same lowercase letter, do not differ.

[a]D40G: 40 days of gestation; D107G: 107 days of gestation; D1L: first day of lactation; D21L: 21 days of lactation.

[b]SEM, standard error of the mean.

Table 5. ARA/EPA ratio at 40 and 107 days of gestation and at 1 and 21 days of lactation of sows

	**Gestation**		**Lactation**	
**Treatment**	**D40G[a] (± SEM[b])**	**D107G[a] (± SEM[b])**	**D1L[a] (± SEM[b])**	**D21L[a] (± SEM[b])**
CON	$63.264 \pm 7.16^{a,A}$	$59.917 \pm 7.59^{a,A}$	$41.825 \pm 3.04^{a,B}$	$25.339 \pm 4.26^{a,C}$
n-6	$70.146 \pm 7.67^{a,A}$	$47.843 \pm 7.40^{a,B}$	$31.797 \pm 2.69^{b,B}$	$31.222 \pm 4.39^{a,B}$
n-3	$64.069 \pm 7.40^{a,A}$	$29.090 \pm 7.59^{b,B}$	$33.114 \pm 2.86^{b,B}$	$23.872 \pm 4.26^{a,B}$

Average on the same line followed by the same capital letter, do not differ. Averages in the same column followed by the same lowercase letter, do not differ.

[a]D40G: 40 days of gestation; D107G: 107 days of gestation; D1L: first day of lactation; D21L: 21 days of lactation.

[b]SEM, standard error of the mean.

Table 6. Backfat thickness average (mm) from 40 to 107 days of gestation of gilts

	**Day**	
**Treatment**	**D40G[a] (± SEM[b])**	**D107G[a] (± SEM[b])**
CON	$15.17 \pm 0.66^{b,A}$	$15.29 \pm 0.45^{a,A}$
n-6	$15.76 \pm 0.66^{b,B}$	$16.41 \pm 0.44^{a,A}$

n-3	17.29 ± 0.56 ^{a,A}	15.44 ± 0.46 ^{a,B}

Average on the same line followed by the same capital letter, do not differ. Averages in the same column followed by the same lowercase letter, do not differ.

[a]D40G: day 40 of gestation; D107G: day 107 of gestation.

[b]SEM, standard error of the mean.

Table 7. Triglyceride's concentration (mg.dL^{-1}) at 40 and 107 days of gestation and at 1 and 21 days of lactation of sows

	**Gestation**		**Lactation**	
**Treatment**	**D40G[a] (± SEM[b])**	**D107G[a] (± SEM[b])**	**D1L[a] (± SEM[b])**	**D21L[a] (± SEM[b])**
CON	129.00 ± 13.92^{a,A}	100.00 ± 13.92^{a,A}	24.10 ± 9.84^{a,B}	100.40 ± 13.92^{a,A}
n-6	95.80 ± 10.81^{a,A}	81.40 ± 10.81^{a,A}	35.33 ± 8.06^{a,B}	67.00 ± 10.81^{b,A}
n-3	80.40 ± 9.69^{b,A}	105.80 ± 9.69^{a,A}	31.11 ± 7.22^{a,B}	54.80 ± 9.69^{b,B}

Average on the same line followed by the same capital letter, do not differ. Averages in the same column followed by the same lowercase letter, do not differ.

[a]D40G: day 40 of gestation; D107G: day 107 of gestation; D1L: day 1 of lactation; D21L: day 21 of lactation.

[b]SEM, standard error of the mean.

Table 8. Effects of supplementing biofortified milk in estimated colostrum production of sows and intake of their piglets in the first 24 hours

	**Treatment[b]**			***P-value*[d]**		
**Item[a]**	**CON (± SEM[c])**	**n-6 (± SEM[c])**	**n-3 ± (SEM[c])**	**Treatment**	**C1**	**C2**
Production, kg	4.867 ± 0.364	4.835 ± 0.393	4.864 ± 0.556	0.998	0.984	0.954
Intake, g	422.943 ± 0.066	409.797 ± 0.070	397.298 ± 0.072	0.827	0.581	0.756

[a]Colostrum intake of piglets alive was estimated by the model to predict of Theil et al. (2014) 24 h after the first-born piglet. The sum of the individual colostrum intake of each piglet within the litter was used to calculate colostrum production of the sow.

[b]Sows fed with a Control milk (CON), supplemented with cow's milk biofortified with n-6 or n-3.

[c]SEM: standard error of the mean.

[d]C1, contrast Control vs n-6+n-3; C2, contrast n-6 vs n-3.

Table 9. Histological characteristics of the *Longissimus dorsi* muscle from gilts at 28 days of gestation – batch B

	**Treatment[b]**			***P- value*[d]**		
**Item[a]**	**CON (± SEM[c])**	**n-6 (± SEM[c])**	**n-3 (± SEM[c])**	**Treatment**	**C1**	**C2**
Adipose cells						
Area, µm	1806.480 ± 226.140	2250.370 ± 225.620	2195.670 ± 214.750	0.321	0.135	0.862
Maximum diameter, µm	63.266 ± 2.897	67.845 ± 2.909	67.819 ± 2.753	0.436	0.200	0.995

Minimum diameter, µm	35.097 ± 2.238	38.107 ± 2.247	38.648 ± 2.127	0.477	0.233	0.863
Medium diameter, µm	47.816 ± 2.475	51.605 ± 2.486	51.763 ± 2.352	0.441	0.204	0.964
Per-area, %	0.220 ± 0.024	0.270 ± 0.024	0.255 ± 0.023	0.327	0.152	0.648
Muscle fiber						
Area, µm	4396.880 ± 737.560	5265.260 ± 755.280	4542.460 ± 780.220	0.413	0.364	0.329
Maximum diameter, µm	88.693 ± 6.062	104.200 ± 6.124	92.680 ± 6.169	0.187	0.197	0.197
Minimum diameter, µm	57.750 ± 4.732	62.934 ± 4.842	58.452 ± 5.001	0.466	0.418	0.352
Medium diameter, µm	72.201 ± 6.200	80.194 ± 6.342	74.028 ± 6.559	0.358	0.297	0.324
Per-area, %	0.345 ± 0.028	0.381 ± 0.028	0.396 ± 0.028	0.429	0.217	0.698

[a]Per-area: percentage of the area filled by the object delimited within the total image, as a way of quantifying the relative area.

[b]Gilts fed with a Control milk (CON), supplemented with cow’s milk biofortified with n-6 or n-3.

[c]SEM: standard error of the mean.

[d]C1, contrast Control vs n-6+n-3; C2, contrast n-6 vs n-3.

Table 10. Histological characteristics of the *Longissimus dorsi* muscle from 14 day-old piglets – batch A

	Treatment[b]			*P-value*[d]				
**Item**[a]	**CON (± SEM**[c]**)**	**n-6 (± SEM**[c]**)**	**n-3 (± SEM**[c]**)**	**Sex**	**Weight**	**Treatment**	**C1**	**C2**
Adipose cells								
Area, µm	841.240 ± 2.760	883.790 ± 2.760	1076.050 ± 2.770	0.006	0.006	0.210	0.245	0.182
Maximum diameter, µm	43.633 ± 2.087	44.638 ± 2.222	48.222 ± 2.494	0.123	0.004	0.265	0.260	0.227
Minimum diameter, µm	25.108 ± 1.432	25.797 ± 1.510	28.841 ± 1.717	0.013	0.004	0.152	0.196	0.134
Medium diameter, µm	33.701 ± 1.722	34.555 ± 1.823	37.808 ± 2.054	0.035	0.003	0.206	0.225	0.182
Per-area, %	0.098 ± 0.029	0.098 ± 0.029	0.114 ± 0.029	0.065	<0.001	0.522	0.580	0.318
Muscle fiber								
Area, µm	250.61 ± 0.10	240.51 ± 0.10	284.72 ± 0.12	0.830	0.002	0.405	0.688	0.192
Maximum diameter, µm	21.606 ± 0.048	21.276 ± 0.049	23.257 ± 0.060	0.980	0.002	0.414	0.637	0.208
Minimum diameter, µm	13.980 ± 0.038	13.776 ± 0.040	14.765 ± 0.048	0.576	0.003	0.448	0.679	0.221
Medium diameter, µm	17.653 ± 0.045	17.262 ± 0.048	18.769 ± 0.056	0.848	0.001	0.427	0.722	0.203
Per-area, %	0.030 ± 0.001	0.029 ± 0.001	0.034 ± 0.001	0.949	0.003	0.444	0.626	0.232

[a]Per-area: percentage of the area filled by the object delimited within the total image, as a way of quantifying the relative area.

[b]Sows fed with a Control milk (CON), supplemented with cow's milk biofortified with n-6 or n-3.

[c]SEM: standard error of the mean.

[d]C1, contrast Control vs n-6+n-3; C2, contrast n-6 vs n-3.

Table 11. Liver expression levels of genes involved in lipid metabolism in gilts from control, n-6 and n-3 groups – batch B

Item	Treatment[a]			*P-value*[c]		
	**TC (± SEM[b])**	**n-6 (± SEM[b])**	**n-3 (± SEM[b])**	**Treat**	**C1**	**C2**
ACC	1.084 ± 0.212	1.049 ± 0.268	1.143 ± 0.212	0.959	0.963	0.786
D6D	1.325 ± 0.326	1.674 ± 0.412	0.966 ± 0.326	0.414	0.991	0.195
FAS	1.308 ± 0.454	1.994 ± 0.525	1.786 ± 0.428	0.589	0.317	0.762
SCD	1.487 ± 0.461	1.114 ± 0.532	1.170 ± 0.435	0.475	0.495	0.358
PPAR-α	1.028 ± 0.123	0.933 ± 0.155	0.889 ± 0.116	0.710	0.462	0.822
SREBP-1	1.131 ± 0.681	2.594 ± 0.786	0.893 ± 0.681	0.235	0.868	0.093

[a]Gilts fed with a Control milk (CON), supplemented with cow's milk biofortified with n-6 or n-3.

[b]SEM: standard error of the mean.

[c]C1, contrast Control vs n-6+n-3; C2, contrast n-6 vs n-3.

Table 12. Relative expression of genes in the gilt hypothalamus supplemented with milk biofortified with PUFA n-6 and n-3

Item	Treatment[a]			*P-value*[c]		
	**TC (± SEM[b])**	**n-6 (± SEM[b])**	**n-3 (± SEM[b])**	**Treat**	**C1**	**C2**
CART	6.198 ± 2.445	3.439 ± 2.445	3.010 ± 2.305	0.986	0.895	0.926
INSR	1.134 ± 0.181	0.637 ± 0.169	1.008 ± 0.159	0.130	0.163	0.125
LEPR	1.842 ± 0.787	1.188 ± 0.736	1.316 ± 0.787	0.794	0.631	0.652
NPY	2.409 ± 0.873	2.211 ± 0.873	2.025 ± 0.823	0.920	0.739	0.812

[a]Gilts fed with a Control milk (CON), supplemented with cow's milk biofortified with n-6 or n-3.

[b]SEM: standard error of the mean.

[c]C1, contrast Control vs n-6+n-3; C2, contrast n-6 vs n-3.

Table 13. Hemolytic activity of the alternative pathway of the complement system for swine females and piglets

Item	Treatment[a]			P-value[c]		
	**CON (± SEM[b])**	**n-6 (± SEM[b])**	**n-3 (± SEM[b])**	**Treat**	**C1**	**C2**
T1/2 Sow batch. A	6.942 ± 0.156	5.966 ± 0.140	4.880 ± 0.156	**<0.001**	**<0.001**	**<0.001**
ΔOD Piglet batch. A	0.088 ± 0.028	0.088 ± 0.028	0.050 ± 0.037	0.680	0.613	0.4343
T1/2 Sow batch. B	6.841 ± 0.31	5.752 ± 0.306	5.774 ± 0.288	**0.028**	**0.008**	0.959

[a]Sows fed with a Control milk (CON), supplemented with cow's milk biofortified with n-6 or n-3.

[b]SEM: standard error of the mean.

[c]C1, contrast Control vs n-6+n-3; C2, contrast n-6 vs n-3.

Table 14. Concentration of IgM (mg.mL^{-1}) at 40, 75 and 107 days of gestation and at 1 and 21 days of lactation of sows

	Gestation			Lactation	
**Treatment**	**D40G[a] (± SEM[b])**	**D75G[a] (± SEM[b])**	**D107G[a] (± SEM[b])**	**D1L[a] (± SEM[b])**	**D21L[a] (± SEM[b])**

CON	15.793 ± 4.123 a,B	14.332 ± 4.123 a,B	16.373 ± 4.123 a,B	29.405 ± 4.123 a,A	20.038 ± 4.123 a,AB
n-6	18.711 ± 3.934 a,A	20.083 ± 3.987 a,A	20.942 ± 4.225 a,A	19.838 ± 3.987 a,A	23.351 ± 3.987 a,A
n-3	15.952 ± 4.123 a,B	14.863 ± 4.123 a,B	17.271 ± 4.123 a,B	17.529 ± 4.123 a,B	27.032 ± 4.123 a,A

Average on the same line followed by the same capital letter, do not differ. Averages in the same column followed by the same lowercase letter, do not differ.

[a]D40G: 40 days of gestation; D75G: 75 days of gestation; D107G: 107 days of gestation; D1L: first day of lactation; D21L: 21 days of lactation.

[b]SEM, standard error of the mean.

Table 15. Concentration of TNF-α (pg.mL^{-1}) at 40, 75 and 107 days of gestation and at 1 and 21 days of lactation of sows

	Gestation			Lactation	
**Treatment**	**D40G[a] (± SEM[b])**	**D75G[a] (± SEM[b])**	**D107G[a] (± SEM[b])**	**D1L[a] (± SEM[b])**	**D21L[a] (± SEM[b])**
CON	32.927 ± 5.376 a,A	30.057 ± 5.376 a,A	37.482 ± 5.376 a,A	22.580 ± 6.191 b,A	22.102 ± 5.376 a,A
n-6	38.544 ± 4.802 a,A	35.048 ± 4.806 a,A	37.403 ± 5.363 a,A	23.122 ± 4.806 b,A	24.078 ± 4.806 a,A
n-3	26.040 ± 5.376 a,B	40.827 ± 5.376 a,AB	29.369 ± 6.191 a,AB	56.531 ± 6.191 a,A	33.560 ± 5.376 a,AB

Average on the same line followed by the same capital letter, do not differ. Averages in the same column followed by the same lowercase letter, do not differ.

[a]D40G: 40 days of gestation; D75G: 75 days of gestation; D107G: 107 days of gestation; D1L: first day of lactation; D21L: 21 days of lactation.

[b]SEM, standard error of the mean.

Table 16. Concentration of IgG (mg.mL^{-1}) at 1 and 14 days of lactation in plasma of piglets

	Day	
**Treatment**	**D1L[a] (± SEM[b])**	**D14L[a] (± SEM[b])**
CON	27.375 ± 17.831 a,B	160.69 ± 18.813 b,A
n-6	25.441 ± 17.831 a,B	262.69 ± 18.813 a,A
n-3	21.867 ± 17.831 a,B	113.65 ± 17.831 c,A

Average on the same line followed by the same capital letter, do not differ. Averages in the same column followed by the same lowercase letter, do not differ.

[a]D1L: 1 day of lactation; D14L: 14 days of lactation.

[b]SEM, standard error of the mean.

Table 17. Concentration of IgM (mg.mL^{-1}) at 1 and 14 days of lactation in plasma of piglets

	Day	
**Treatment**	**D1L[a] (± SEM[b])**	**D14L[a] (± SEM[b])**
CON	2.988 ± 0.940 a,B	6.358 ± 0.992 b,A
n-6	0.812 ± 0.940 b,B	10.295 ± 0.992 a,A
n-3	0.989 ± 0.940 b,B	7.395 ± 0.992 ab,A

Average on the same line followed by the same capital letter, do not differ. Averages in the same column followed by the same lowercase letter, do not differ.

[a]D1L: 1 day of lactation; D14L: 14 days of lactation.

[b]SEM, standard error of the mean

Table 18. Plasma concentrations of LTB4, PGF2α and TXB2 in sows at 13 ± 4 days of lactation

**Item[a]**	**Treatment[b]**					***P-value*[d]**				
	**CON (± SEM[c])**	**n-6 (± SEM[c])**	**n-3 (± SEM[c])**	**TAP**	**DMSO**	**Treat**	**Stimulus**	**Treat*Sti**	**C1**	**C2**
LTB4, pg.mL^{-1}	580.281 ± 213.378	647.393 ± 197.550	448.515 ± 261.334	558.729 ± 130.321	n.e.	0.879	n.e.	n.e.	0.715	0.723
PGF2α, pg.mL^{-1}	46.911 ± 36.291	57.158 ± 28.111	41.504 ± 38.936	72.363 ± 19.754	n.e.	0.390	0.063	0.302	n.e.	0.463
TXB2, pg.mL^{-1}	20.266 ± 24.119	38.421 ± 26.554	58.128 ± 43.023	74.793 ± 18.777	3.084 ± 32.280	0.847	0.0001	0.922	0.908	0.580

[a]Analytes present in greater evidence in the sample

[b]Sows fed with a Control milk (CON), supplemented with cow's milk biofortified with n-6 or n-3.

[c]SEM: standard error of the mean.

[d]C1, contrast Control vs n-6+n-3; C2, contrast n-6 vs n-3.

n.e: not estimable. The values were not estimable due to missing, as a consequence of the limitations of the method to measure the concentration of analytes in the sample.

Table 19. Plasma concentrations of LTB4, PGF2α and TXB2 in piglets at 13 ± 4 days of age

**Item[a]**	**Treatment[b]**					***P-value*[d]**				
	**CON (± SEM[c])**	**n-6 (± SEM[c])**	**n-3 (± SEM[c])**	**TAP**	**DMSO**	**Treat**	**Stimulus**	**Treat*Sti**	**C1**	**C2**
LTB4, pg.mL^{-1}	257.400 ± 205.240	498.349 ± 229.466	568.989 ± 458.931	441.579 ± 184.209	n.e.	0.625	n.e.	n.e.	0.472	0.551
PGF2α, pg.mL^{-1}	48.433 ± 126.229	278.644 ± 105.937	75.158 ± 180.405	147.565 ± 86.231	n.e.	0.913	0.176	n.e.	n.e.	n.e.
TXB2, pg.mL^{-1}	45.245 ± 6.095	31.884 ± 5.220	76.663 ± 7.869	88.2901 ± 5.502	14.238 ± 5.086	0.608	<0.0001	0.094	0.347	0.796

[a]Analytes present in greater evidence in the sample

[b]Sows fed with a Control milk (CON), supplemented with cow's milk biofortified with n-6 or n-3.

[c]SEM: standard error of the mean.

[d]C1, contrast Control vs n-6+n-3; C2, contrast n-6 vs n-3.

n.e: not estimable. The values were not estimated due to missing, as a consequence of the limitations of the method to measure the concentration of analytes in the sample.

Table 20. Plasma concentrations of LTB4, PGF2α and TXB2 in gilts at 24 ± 6 days of gestation

**Item**[a]	**Treatment**[b]					***P-value***[d]				
	**CON (± SEM**[c]**)**	**n-6 (± SEM**[c]**)**	**n-3 (± SEM**[c]**)**	**TAP**	**DMSO**	**Trat**	**Stimulus**	**Treat*Sti**	**C1**	**C2**
LTB4, pg.mL^{-1}	240.085 ± 324.069	330.581 ± 330.016	129.152 ± 322.062	403.904 ± 129.271	62.641 ± 352.802	0.465	0.037	0.784	0.359	0.414
PGF2α, pg.mL^{-1}	26.425 ± 18.191	72.973 ± 18.191	22.171 ± 24.255	31.180 ± 9.902	49.866 ± 21.391	0.403	0.890	0.110	0.960	0.183
TXB2, pg.mL^{-1}	11.924 ± 4.755	7.042 ± 4.842	16.615 ± 3.381	19.852 ± 2.606	3.8685 ± 4.331	0.139	<0.0001	0.550	0.114	0.212

[a]Analytes present in greater evidence in the sample.

[b]Gilts fed with a Control milk (CON), supplemented with cow's milk biofortified with n-6 or n-3.

[c]SEM: standard error of the mean.

[d]C1, contrast Control vs n-6+n-3; C2, contrast n-6 vs n-3.
