# Supplementary material for: Feeding sows milk biofortified with n-6 and n-3 modulates immune status of sows and drives positive transgenerational effects
Source: PLoS One. 2024 Aug 27;19(8):e0306707. doi: 10.1371/journal.pone.0306707 (PMC11349115; doi:10.1371/journal.pone.0306707)
Supplement: S2 File — (PDF) [file pone.0306707.s002.pdf]

Feeding sows milk biofortified with n-6 and n-3 modulates immune status of sows and drives positive transgenerational effects

Authors: Leriana Garcia Reis[1*,#a], Vera Letticie de Azevedo Ruiz[2&], Simone Maria Massami Kitamura[3], André Furugen Cesar Andrade[4], Fernando de Oliveira Bussiman[5], Mirele Daiana Poleti[2], Juliano Coelho da Silveira[2], Heidge Fukumasu[2], Lúcia Helena Faccioli[6], Cleni Mara Marzocchi-Machado[6], Ricardo de Francisco Strefezzi[2], Edna Neves Garcia[3], Theresa Casey[1], Arlindo Saran Netto[3,#b].

Short Tittle: Biofortified Milk's Immune Modulation in Sows and Transgenerational Benefits with n-6 and n-3

[1]Department of Animal Science, Purdue University, West Lafayette, Indiana, USA.

[2]Department of Veterinary Medicine, School of Animal Science and Food Engineering, University of São Paulo, Pirassununga, SP, Brazil.

[3]Department of Animal Science, School of Animal Science and Food Engineering, University of São Paulo

[4]Department of Animal Reproduction, School of Animal Science and Food Engineering, University of São Paulo

[5]Department of Animal and Dairy Science, University of Georgia, Athens Georgia , USA.

[6]Department of Clinical Analyses, Toxicology and Food Sciences, School of Pharmaceutical Sciences of Ribeirão Preto, University of São Paulo, Ribeirão Preto, SP, Brazil.

[#a]Current address: Department of Animal Science, Purdue University, West Lafayette, Indiana 47907, USA

[#b]Current address: Department of Animal Science, School of Animal Science and Food Engineering, University of São Paulo, Rua Duque de Caxias Norte, 225, 13635-900, Pirassununga, SP, Brazil

*Corresponding author:

Email: lgarciar@purdue.edu/lerianagarcia@outlook.com (L.G.R.)

Key words: Essential fatty acids. Immune modulation. Maternal nutrition. Piglets. Swine reproductive outcomes.

Pre-initial - UNIMIX 01.541 (guaranteed levels per kilogram of product): acetic acid (min) 1,500.0 mg; benzoic acid (min) 750.0 mg; folic acid (min) 0.9 mg; formic acid (min) 2,850.0 mg; phosphoric acid (min) 315.0 mg; pantothenic acid (min) 40 mg; alpha-galactosidase (min) 17.5 U; Bacillus subtilis (min) 7.5 x 108 CFU; betaglucanase (min) 550.0 U; biotin (min) 0.33 mg; calcium (min - max) 12.5-1.0 g; copper (min) 25.0 mg; choline (min) 855 mg; colistin 98.0 mg; ether extract (min) 65.0 g; iron (min) 205.0 mg; crude fiber (max) 13.5 mg; phytase (min) 1,250.0 FTU; phosphorus (min) 9,600.0 mg; galactomannanase (min) 55.0 U; iodine (min) 2.5 mg; lysine (min) 19.0 g; manganese (min) 90 mg; ash (max) 100.0 g; methionine (min) 9,000.0 mg; niacin (min) 70.0 mg; crude protein (min) 140.0 g; selenium (min) 0.8 mg; sodium (min) 7,300.0 mg; threonine (min) 10.0 g; tryptophan (min) 2,500.0 mg; moisture (max) 100.0 g; vitamin A (min) 25,000.0 IU; vitamin B1 (min) 2.7 mg; vitamin B12 (min) 55.0 mcg; vitamin B2 (min) 10.0 mg; vitamin B6 (min) 5.0 mg; vitamin D3 (min) 4,000.0 IU; vitamin E (min) 135.0 IU; vitamin K3 (min) 4.5 mg; xylanase (min) 750 U; zinc (min) 5,500.0 mg.

Initial - UNIMIX 01.592 (guarantee levels per kilogram of product): folic acid (min) 0.4 mg; pantothenic acid (min) 206.0 mg; Bacillus subtilis (min) 6.0 x 10^9 CFU; biotin (min) 1.0 mg; calcium (min - max) 100.0-130.0 g; copper (min) 2,989.0 mg; choline (min) 1,500.0 mg; ether extract (min) 19.6 g; iron (min) 1,620.0 mg; crude fiber (max) 16.3 g; phytase (min) 12,000.0 FTU; phosphorus (min) 32.0 g; halquinol 2,400.0 mg; iodine (min) 19.0 mg; lysine (min) 16.8 g; manganese (min) 723.0 mg; mineral matter (max) 197.5 g; niacin (min) 296.0 mg; crude protein (min) 23.6 g; selenium (min) 6.4 mg; sodium (min) 38.0 g; vitamin A (min) 200,000.0 IU; vitamin B1 (min) 39.9 mg; vitamin B12 (min) 399.0 mcg; vitamin B2 (min) 79.0 mg; vitamin B6 (min) 29.9 mg; vitamin D3 (min) 40,000.0 IU; vitamin E (min) 400.0 IU; vitamin K3 (min) 40.0 mg; zinc (min) 2,100.0 mg.

Growth - UNIMIX 01.555 (guaranteed levels per kilogram of product): folic acid (min) 9.6 mg; pantothenic acid (min) 410.2 mg; biotin (min) 3.9 mg; calcium (min - max) 173.6-250.4 g; copper (min) 5,964.7 mg; choline (min) 7,569.0 mg; iron (min) 1,606.6 mg; phytase (min) 30,000.0 FTU; phosphorus (min) 46.6 g; iodine (min) 75.9 mg; lysine (min) 8.4 g; manganese (min) 3,536.2 mg; mineral matter (max) 374.7 g; niacin (min) 590.4 mg; selenium (min) 14.0 mg; sodium (min) 64.1 g; vitamin A (min) 400,000.0 IU; vitamin B1 (min) 79.5 mg; vitamin B12 (min) 795.2 mcg; vitamin B2 (min) 158.9 mg; vitamin B6 (min) 59.6 mg; vitamin D3 (min) 80,000.0 IU; vitamin E (min) 800.0 IU; vitamin K3 (min) 81.6 mg; zinc (min) 5,358.1 mg.

Finisher and Replacement - UNIMIX 01.556 (guaranteed levels per kilogram of product): folic acid (min) 7.3 mg; pantothenic acid (min) 310.5 mg; biotin (min) 3.0 mg; calcium (min - max) 175.9-263.9 g; copper (min) 5,693.9 mg;

choline (min) 4,875.5 mg; iron (min) 1,603.7 mg; phytase (min) 30,000.0 FTU; phosphorus (min) 23.7 g; iodine (min) 75.9 mg; lysine (min) 1.6 g; manganese (min) 3,529.8 mg; mineral matter (min) 294.2 g; niacin (min) 447.0 mg; selenium (min) 12.6 mg; sodium (min) 61.7 g; vitamin A (min) 250,000.0 IU; vitamin B1 (min) 60.2 mg; vitamin B12 (min) 602.1 mcg; vitamin B2 (min) 120.3 mg; vitamin B6 (min) 45.2 mg; vitamin D3 (min) 50,000.0 IU; vitamin E (min) 500.0 IU; vitamin K3 (min) 51.0 mg; zinc (min) 5,348.4 mg.

Gestation - UNIMIX 01.550 (guarantee levels per kilogram of product): folic acid (min) 41.8 mg; pantothenic acid (min) 482.2 mg; biotin (min) 4.0 mg; calcium (min - max) 261.6-261.6 g; copper (min) 4,729.9 mg; choline (min) 10.2 g; iron (min) 1,155.8 mg; crude fiber (max) 0.2 g; phytase (min) 20,000.0 FTU; phosphorus (min) 33.9 g; iodine (min) 19.5 mg; lysine (min) 5.0 g; manganese (min) 2,544.0 mg; mineral matter (max) 231.0 g; niacin (min) 803.9 mg; crude protein (min) 5.9 g; selenium (min) 12.0 mg; sodium (min) 63.2 g; moisture (max) 100.0 g; vitamin A (min) 420,000.0 IU; vitamin B1 (min) 101.9 mg; vitamin B12 (min) 600.0 mcg; vitamin B2 (min) 160.0 mg; vitamin B6 (min) 62.7 mg; vitamin D3 (min) 60,000.0 IU; vitamin E (min) 1,200.0 IU; vitamin K3 (min) 61.2 mg; zinc (min) 3,854.7 mg.

Lactation - UNIMIX 01.551 (guaranteed levels per kilogram of product): folic acid (min) 51.2 mg; pantothenic acid (min) 590.6 mg; amylase (min) 4,800.0 U; betaglucanase (min) 8,400.0 U; biotin (min) 4.9 mg; calcium (min - max) 212.9-319.3 g; cellulase (min) 72,000 U; copper (min) 4,487.9 mg; choline (min) 8,352.0 mg; iron (min) 1,040.3 mg; phytase (min) 13,200.0 FTU; phosphorus (min) 41.4 g; iodine (min) 17.6 mg; lysine (min) 3.9 g; manganese (min) 2,289.6 mg; niacin (min) 984.8 mg; protease (min) 8,400.0 U; crude protein (min) 3.8 g; selenium (min) 14.6 mg; sodium (min) 41.2 g; moisture (min) 100.0 g; vitamin A (min) 514,500.0 IU; vitamin B1 (min) 124.9 mg; vitamin B12 (min) 735.0 mcg; vitamin B2 (min) 196.0 mg; vitamin B6 (min) 76.8 mg; vitamin D3 (min) 73,500.0 IU; vitamin E (min) 1,470.0 IU; vitamin K3 (min) 75.0 mg; xylanase (min) 120,000 U; zinc (min) 3,469.2 mg.
